# The Key Factors in Physical Activity Type Detection Using Real-Life Data: A Systematic Review

**DOI:** 10.3389/fphys.2019.00075

**Published:** 2019-02-12

**Authors:** Hoda Allahbakhshi, Timo Hinrichs, Haosheng Huang, Robert Weibel

**Affiliations:** ^1^Geographic Information Systems Unit, Department of Geography, University of Zurich (UZH), Zurich, Switzerland; ^2^Division of Sports and Exercise Medicine, Department of Sports, Exercise and Health, University of Basel, Basel, Switzerland

**Keywords:** physical activity type, real-life, accelerometer, sensor, systematic review

## Abstract

**Background:** Physical activity (PA) is paramount for human health and well-being. However, there is a lack of information regarding the types of PA and the way they can exert an influence on functional and mental health as well as quality of life. Studies have measured and classified PA type in controlled conditions, but only provided limited insight into the validity of classifiers under real-life conditions. The advantage of utilizing the type dimension and the significance of real-life study designs for PA monitoring brought us to conduct a systematic literature review on PA type detection (PATD) under real-life conditions focused on three main criteria: methods for detecting PA types, using accelerometer data collected by portable devices, and real-life settings.

**Method:** The search of the databases, Web of Science, Scopus, PsycINFO, and PubMed, identified 1,170 publications. After screening of titles, abstracts and full texts using the above selection criteria, 21 publications were included in this review.

**Results:** This review is organized according to the three key elements constituting the PATD process using real-life datasets, including data collection, preprocessing, and PATD methods. Recommendations regarding these key elements are proposed, particularly regarding two important PA classes, i.e., posture and motion activities. Existing studies generally reported high to near-perfect classification accuracies. However, the data collection protocols and performance reporting schemes used varied significantly between studies, hindering a transparent performance comparison across methods.

**Conclusion:** Generally, considerably less studies focused on PA types, compared to other measures of PA assessment, such as PA intensity, and even less focused on real-life settings. To reliably differentiate the basic postures and motion activities in real life, two 3D accelerometers (thigh and hip) sampling at 20 Hz were found to provide the minimal sensor configuration. Decision trees are the most common classifier used in practical applications with real-life data. Despite the significant progress made over the past year in assessing PA in real-life settings, it remains difficult, if not impossible, to compare the performance of the various proposed methods. Thus, there is an urgent need for labeled, fully documented, and openly available reference datasets including a common evaluation framework.

## Introduction

Physical activity (PA) is a key component of promoting health and well-being (Haskell et al., 2007), and “is defined as any bodily movement produced by skeletal muscles that results in energy expenditure” (Caspersen et al., 1985). A physically active lifestyle is crucial for healthy aging and is associated with several important health outcomes, such as higher levels of functional health, a lower risk of falling, and better cognitive function (Voss et al., 2016). PA is a complex behavior with four main dimensions, which can be abbreviated as FITT: Frequency of the activity, usually measured in occasions per week; Intensity at which the activity is carried out; Time: the duration of the bout of activity; and Type of activity (Cavill et al., 2006).

This paper aims to systematically review the existing methodologies that meet the three main criteria: (1) they detect *PA types*; (2) the PA data collection is performed in *real-life settings*; and (3) portable devices used include *accelerometer sensors* (and possibly additional sensors).

### PA Types

Researchers utilize various subjective and objective techniques as well as PA dimensions (FITT) to characterize and interpret PA. There are numerous studies using thresholds to derive *PA intensity* (or *PA level*, i.e., categorized PA intensity) to quantify human PA (Clemente et al., 2016; Vanroy et al., 2016; Krüger et al., 2017; Laakkonen et al., 2017; McCarthy et al., 2017; Rockette-Wagner et al., 2017; Vanderloo and Tucker, 2017). However, it is controversial whether defined thresholds for assessing PA levels can be applied to people from different age groups. Most studies measuring PA in everyday life have focused on *energy expenditure*, but there are studies that report that PA types per se, such as walking and sitting, can influence health (Hamer and Chida, 2007; Patel et al., 2010). There is evidence that suggests *self-reported measures* underestimate postural positions and overestimate postural duration (Unge et al., 2005; Teschke et al., 2009), hence objective, reproducible methods providing valid measurements of PA types during daily life are required (Unge et al., 2005; Hendrick et al., 2009).

Today, little is known about *types* and patterns of PA and the way they can influence functional and mental health as well as quality of life (Taraldsen et al., 2012). Accurate measurement of the daily PA types independently of other PA measures is therefore important, with one of the challenges in PA research being to quantify exactly how much and what type of PA is taking place (McCarthy and Grey, 2015). Furthermore, the concept of PA types is easier to communicate. For instance, recommendations regarding the amount of time that should be spent walking are easier to follow than recommending a certain level of activity intensity, a concept that most laypersons are unlikely to clearly understand. Understanding how a specific type of PA, such as walking, can play a role in human health, providing useful guidance that is more tangible for people. It would further facilitate a more transparent comparison of accelerometer data across different studies (Hendrick et al., 2009).

### Real-Life Settings

Most studies reporting on the development of measurement methods to classify PA types have used an experimental protocol based on predefined physical activities under laboratory, that is, controlled conditions (van Hees et al., 2013). However, laboratory settings invariably separate participants from their everyday life and activities, subjecting them to an artificial context (Csikszentmihalyi, 2011). It is thus questionable whether and to what extent laboratory-derived algorithms and models can be applied to data acquired under real-life conditions (De Vries et al., 2011). Natural daily settings such as home, workplace, school, or outdoor environments are examples of real-life conditions. Experimental protocols performed under controlled conditions are commonly used for the training and evaluation of PA type classification techniques. Such protocols, however, only offer limited insight into the validity of PA classification under real-life conditions (van Hees et al., 2013). Therefore, automatic PA type detection methods should be developed using data acquired in real-life conditions (Bastian et al., 2015). Assessment of PA outside a laboratory setting is important because people's daily activity is typically different from what can be measured in the clinic (Hache et al., 2011).

### Accelerometer Sensors

Sensors are the main sources for PA type recognition. Among the existing portable sensors, accelerometers have gained most attention and are increasingly popular among users (Shoaib et al., 2014). Accelerometers are small, light-weight, and mobile sensors that can record acceleration as well as information about the movement and activity of users. Accelerometers may be built into custom-made activity sensing devices, but current smartphones also include built-in accelerometers and related sensors, which further enhances the potential of such sensors for PA measurement.

Recently, several review articles have discussed PA from different perspectives, with some focused on a particular age group (Murphy, 2009; Taraldsen et al., 2012; Schrack et al., 2016). As noted by Taraldsen et al. (2012) and Schrack et al. (2016), a great variety of methods exist for collecting and analyzing PA data, which poses significant challenges to comparing and synthesizing results. There is an urgent need for establishing guidelines for analyzing and interpreting PA data. Preece et al. (2009) and Clark et al. (2017) reviewed the literature with a focus on analysis techniques used for monitoring PA. There are also review papers highlighting the data collection step in an attempt to provide information about how different devices can be utilized in the PA monitoring process (McCarthy and Grey, 2015; Cornacchia et al., 2017; Migueles et al., 2017).

Despite the series of existing review articles, we believe that it is timely to conduct a systematic review of the literature in PA monitoring with a focus on *methods of PA type detection (PATD) in real-life environments using accelerometer measurements*, which to the best of our knowledge does not exist so far. There are still gaps in the existing related reviews that warrant an updated synthesis and more information is required about methodological issues using real-life data for PATD. By systematically analyzing and comparing the related literature, we seek to provide relevant insights to help researchers select appropriate study designs and data processing methods when conducting a study using accelerometers for PA type detection.

## Methodology

This section introduces the research questions and the methodology adopted in this paper.

### Research Questions

This review paper aims to answer the following research questions:

Q1: What are the characteristics of commonly used data collection processes for PATD? The data collection processes will be compared according to ambulatory assessment specifications and participant characteristics.
Q2: What are the existing preprocessing methods used in real-life PATD with respect to segmentation, as well as feature extraction and selection? The review will mainly focus on the strengths and weaknesses of these methods and the signal features that are commonly used.
Q3: What methods are used for physical activity type detection, and what are their strengths and weaknesses? These methods will be compared according to accuracy, number, and types of activities detected, as well as the size of the training dataset.

### Study Selection

Four databases were searched to conduct the systematic literature review, including Web of Science, Scopus, PsycINFO, and MEDLINE (PubMed) using keywords contained in the title, abstract, keywords, text, and topic. The selection of databases, keywords, and inclusion criteria was based on extensive research in the PA area and brainstorming with experts. This paper considers post-1990 literature, as to the best of our knowledge, PATD in real-life was first introduced in 1996 (Makikawa and Murakami, 1996). Two categories of search terms were used and at least one of each two categories of search terms must be used to combine: (1) “Acceleromet^*^,” “Inertial measurement unit,” “gyroscope,” “IMU”; (2) (“Physical activit^*^” and ((“mode”) or (“type”))).

### Inclusion and Exclusion Criteria

The inclusion criteria were: (1) focus on methods for PA type detection; (2) data collection in real-life conditions; (3); one or more portable devices used including accelerometer sensor(s) to collect PA-related data; and (4) written in English. When there were multiple papers from the same authors presenting the same methodology, only the most comprehensive article was included in the review. Papers reporting outdoor data collection in addition to laboratory-based data collection were also included. Studies not reporting additional information about PATD methods, which means not making some improvements or meaningful comparison of existing methods, were excluded. Book chapters, dissertations, review papers, and studies using non-human data were also excluded. The first paper introducing the concept of PATD in real-life (Makikawa and Murakami, 1996) was included despite it lacking a practical section.

**Figure 1:**
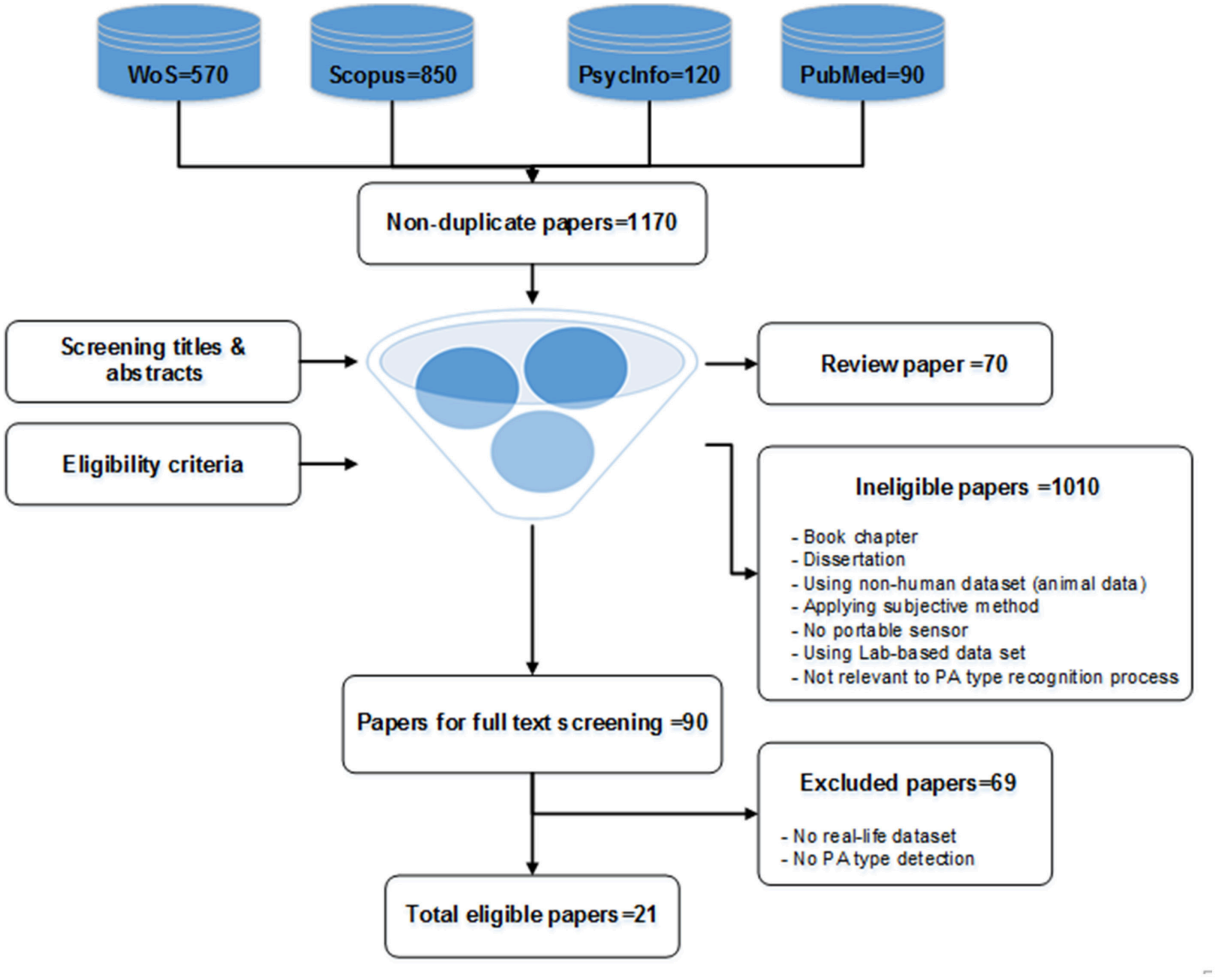
Flowchart of the systematic literature review on PATD.

The search process is illustrated in Figure 1. The number of papers collected from each database were: 570 (Web of Science), 850 (Scopus), 120 (PsycINFO), 90 (PubMed). After removing duplicates, 1,170 were identified, of which 90 were retained following the screening of titles and abstracts. After full text screening, 21 publications were included in the detailed review. The first author assessed all titles and abstracts and all full text articles, decisions to accept or reject a paper were agreed among all authors.

### Data Extraction

**Figure 2:**
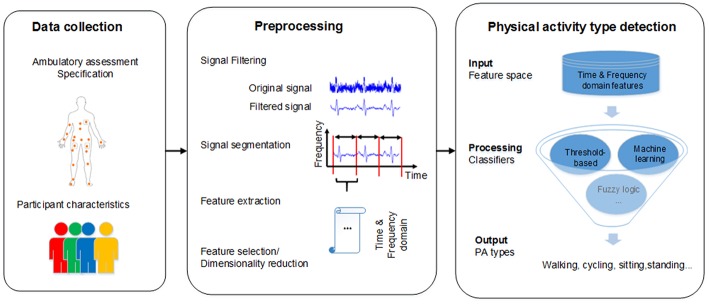
Flowchart of PATD process.

The literature review was organized according to the three main stages of the PATD process: data collection, preprocessing, and PATD. As shown in Figure 2, various factors can play a role in how accurately a PA type can be detected using real-life data. Thus, the information extracted from each article relates to these key factors, providing the structure for reporting results:

- Data collection (Data Collection): ambulatory assessment specification (e.g., device type, sampling rate); participant characteristics (e.g., number of participants, age group)
- Data preprocessing methods (Section Preprocessing Methods): signal filtering; signal segmentation; feature extraction; feature selection/dimensionality reduction
- PATD methods (Physical Activity Type Detection Methods): focusing on different PATD classifiers as well as how they compare regarding the classification of different PA types.

## Results

### Data Collection

This section organizes the results regarding data collection and attempts to answer the first research question. The extracted information is summarized in Tables 1–**3**. Some studies did not report device specifications or participant characteristics, and thus were not included in the tables.

**Table 1:** Ambulatory assessment specification.

**Ambulatory assessment specification**			**References**
Device	Customized device (e.g., **Actigraph**, Tracmor, IDEEA, etc.)		**Troped et al., 2008; De Vries et al., 2011; Ruch et al., 2011; Skotte et al., 2014; Fergus et al., 2015,** Bonomi et al., 2009; Godfrey et al., 2011; Gyllensten and Bonomi, 2011; Reiss and Stricker, 2011; Kwak and Lee, 2012; Nguyen et al., 2013; van Hees et al., 2013; Adaskevicius, 2014; Barshan and Yuksek, 2014; el Achkar et al., 2016
	Smartphone		Bisio et al., 2012; Bayat et al., 2014; Shoaib et al., 2014; Spinsante et al., 2016
Sensor type	Accelerometer **Accelerometer + Additional sensor** (e.g., heart rate, GPS, pressure sensor, imaging sensor, barometer, etc.)	1D	De Vries et al., 2011, **Troped et al., 2008; Ruch et al., 2011; Nguyen et al., 2013; Fergus et al., 2015; el Achkar et al., 2016**
		2D	Nguyen et al., 2013; el Achkar et al., 2016
		3D	Bonomi et al., 2009; Godfrey et al., 2011; Gyllensten and Bonomi, 2011; Bisio et al., 2012; Bayat et al., 2014; Spinsante et al., 2016, **Kwak and Lee, 2012; van Hees et al., 2013; Adaskevicius, 2014; Shoaib et al., 2014; Skotte et al., 2014**
	IMU (3D accelerometer + 3D gyroscope + 3D magnetometer) **IMU+ Additional sensor**		Barshan and Yuksek, 2014, **Reiss and Stricker, 2011; el Achkar et al., 2016**
Number of sensors	1 Accelerometer **1 Accelerometer + Additional Sensor**		Bonomi et al., 2009; Godfrey et al., 2011; Bisio et al., 2012; Bayat et al., 2014; Spinsante et al., 2016, **Troped et al., 2008; Kwak and Lee, 2012; Adaskevicius, 2014; Fergus et al., 2015**
	>1 Accelerometer **>1 Accelerometer + Additional Sensor**		De Vries et al., 2011, **Reiss and Stricker, 2011; Ruch et al., 2011; Nguyen et al., 2013; van Hees et al., 2013; Barshan and Yuksek, 2014; Shoaib et al., 2014; Skotte et al., 2014; el Achkar et al., 2016**
Sampling rate	Counts/steps		Troped et al., 2008; De Vries et al., 2011; Godfrey et al., 2011; Ruch et al., 2011; Nguyen et al., 2013; Fergus et al., 2015; el Achkar et al., 2016
	Medium (20–50 Hz)		Bonomi et al., 2009; Gyllensten and Bonomi, 2011; Adaskevicius, 2014; Barshan and Yuksek, 2014; Shoaib et al., 2014 Skotte et al., 2014 Garcia-Ceja and Brena, 2016 Spinsante et al., 2016
	High (>50 Hz)		Reiss and Stricker, 2011; van Hees et al., 2013; Bayat et al., 2014; Garcia-Ceja and Brena, 2016
Sensor placement	One part of body	C	Makikawa and Murakami, 1996; Troped et al., 2008; Bonomi et al., 2009; Fergus et al., 2015
		U	Godfrey et al., 2011; Kwak and Lee, 2012; Adaskevicius, 2014
		L	Bisio et al., 2012; Spinsante et al., 2016
	Two parts of body	H&L	Bayat et al., 2014
		C&L	De Vries et al., 2011; Nguyen et al., 2013; Skotte et al., 2014; el Achkar et al., 2016
		C&H	Ruch et al., 2011
	Three parts of body	U&C&L	Gyllensten and Bonomi, 2011
		U&L&H	Reiss and Stricker, 2011; Barshan and Yuksek, 2014
		C&L&H	van Hees et al., 2013; Shoaib et al., 2014
	Four parts of body	C&L&H&U	Garcia-Ceja and Brena, 2016

*D, Dimension; IMU, inertial measurement unit; Hz, Hertz; C, central part of body; U, upper part of body; L, lower part of body; H, hand*.

#### Ambulatory Assessment Specification

The ambulatory assessment specification includes information about the type of device and sensors used for the data collection, the sampling rate, and sensor placement.

##### Device type

**Figure 3:**
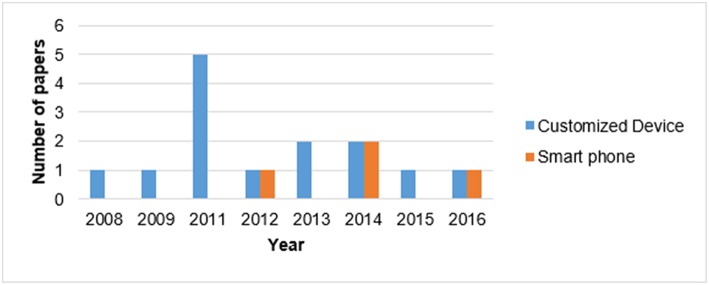
Temporal trend of the devices used for PATD.

Monitoring PA in real-life has seen major advances in the past decade due to progress made in mobile and wearable technology. There are important factors such as availability, cost, and wearing comfort for the users, sensor specifications of the measurement device and the target physical activities being identified, all of which can play a role in choosing an appropriate device for the study. The device used for the data collection is the cornerstone of the PATD process, as it contains all the necessary information, such as sensor specification and raw data, on which the preprocessing and data analysis are based. Different devices have different technical specifications, leading to data with different characteristics. On one hand, it is possible to have several sensors embedded in a single device, yielding a “multi-sensor configuration.” On the other hand, in a “multi-device configuration,” several devices are used on different parts of the body. As shown in Table 1, the existing customized or commercial devices for objective movement analysis (e.g., Actigraph, Tracmor, INEEA, etc.) as well as smartphones have the potential to be used for PATD. Among them, customized devices, particularly the Actigraph (Actigraph LLC., Pensacola, FL), which enables daily recordings for several days (Skotte et al., 2014), is the most common device type used for data collection. The papers using the Actigraph are highlighted in bold in Table 1. For a comprehensive review of existing studies assessing PA using the ActiGraph GT3X, please refer to (Migueles et al., 2017). As shown in Figure 3, there has been a recent trend towards using smartphones. Smartphones are the seconds most common device type used for PATD, since they feature multiple sensors. Also, there is no need for the participant to carry an additional device, which leads to less annoyance in long-term, real-life PA monitoring. However, using several sensors simultaneously causes more battery consumption, which can be problematic for a battery-limited device, such as a smartphone. Moreover, sampling may be interrupted or rates slowed down if priority services (such as an incoming phone call) take precedence.

##### Sensor type

During the past decade, the tendency to utilize mobile sensing in PATD increased dramatically, owing to wearable sensors for continuous daily PA monitoring of subjects. Different sensors can obtain different movement information, informing the classifiers used for PA type recognition. Our results indicate that in addition to accelerometers, there were various additional portable sensors, termed “additional sensors,” such as heart rate sensors (Kwak and Lee, 2012; Fergus et al., 2015), barometer and foot pressure sensors (Skotte et al., 2014; el Achkar et al., 2016), imaging sensors (Ruch et al., 2011; van Hees et al., 2013; Adaskevicius, 2014), and GPS (Troped et al., 2008; Nguyen et al., 2013). For example, in el Achkar et al. (2016) a gyroscope helped to differentiate between motion and posture states. Foot pressure sensors provided information to separate sitting from standing. Barometers were used for identification of activities with elevation change, and finally accelerometer data were utilized for recognizing stairs and ramp climbing. In Kwak and Lee (2012), combining heart rate data with accelerometer data increased the fuzzy classification performance by 20% for differentiating walking speeds compared to using an accelerometer alone. The studies using accelerometers and additional sensors are highlighted in bold in Table 1. Commonly, these sensors are all easy-to-use, cheap, and small enough to avoid putting a prohibitive burden on the participant. Multi-sensor configurations provide better results for activity classification but are not suitable for long-term monitoring if all the sensors are not embedded in a single device, which in turn raises an important issue regarding sensor placement (el Achkar et al., 2016).

**Figure 4:**
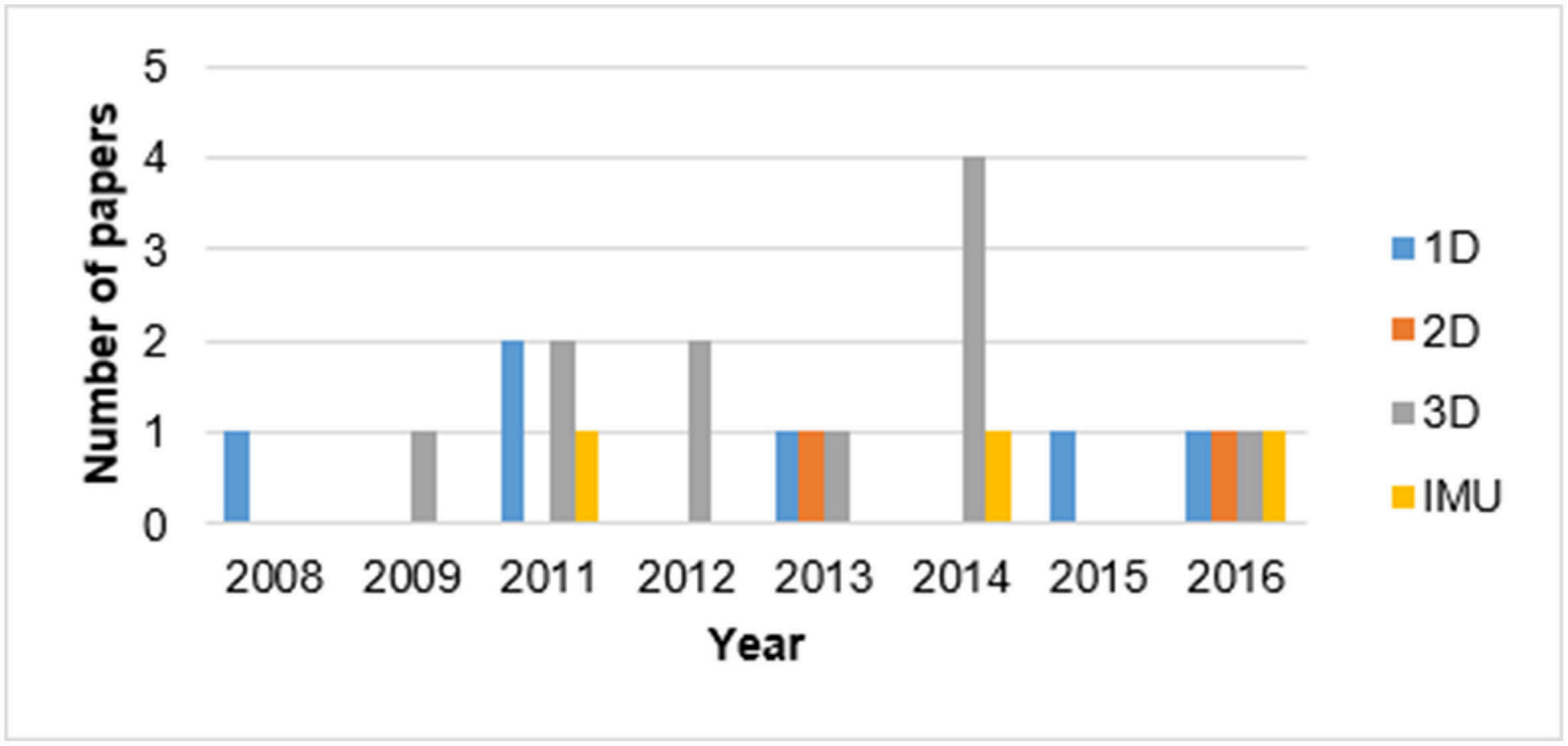
Temporal trend of the different types of accelerometers used for PATD.

Among the existing wearable sensors, the accelerometer has gained the most attention and increasing popularity among users (Shoaib et al., 2014). There are different types of accelerometer, including uni-axial (1D), dual-axial (2D), and tree-axial (3D). Results indicated that using data from multiple-axial accelerometers improves the accuracy of PATD models. However, in recent years, other sensors, such as the gyroscope and magnetometer, have been combined with an accelerometer to build inertial measurement units (IMU) to improve activity recognition performance (Shoaib et al., 2014) (Figure 4). As 3D accelerometers can provide data in three different dimensions, more distinctive features can be extracted compared to when using 1D or 2D accelerometers, respectively. Figure 4 shows that more than 50% of the studies tended to use 3D accelerometers, followed by 1D-accelerometers, 3D-IMU, and 2D-accelerometers, respectively.

##### Number of sensors

Determining the number of sensors to use for real-life data collection is challenging. Increasing the number of sensors may raise the number of activities that can be classified and improve the classification performance, but may also render the data analysis more complex and place more burden on participants if it requires a multi-device configuration. Conversely, using only a single accelerometer may not provide enough information for detecting different types of activity. For example, De Vries et al. (2011) show that dual accelerometer placement (hip and ankle) should be considered when detecting activities such as sitting, standing, using the stairs, walking, and cycling rather than using only one hip-worn accelerometer. This is supported by Gyllensten and Bonomi (2011), who also found that it is problematic to classify real-life activity data by laboratory-trained algorithms using only a single waist-mounted 3D accelerometer. Conversely, studies have shown that using a single sensor can lead to meaningful results in PATD if the appropriate signal features are chosen (Bayat et al., 2014; Shoaib et al., 2014). In general, the number and kinds of PA types targeted for detection can also play role in choosing the number of sensors for data collection. Approximately 50% of the included studies utilized only one accelerometer for the PATD and achieved reasonable results for distinguishing postures and motion activities. Nonetheless, as mentioned above some studies applied additional sensors (highlighted in bold in Table 1).

##### Sampling rate

The sampling rate is the number of readings of accelerometer data recorded per unit time. The sampling rate used for PATD varied between 10 and 100 Hz. Some studies also applied the terms “activity count” (Troped et al., 2008; De Vries et al., 2011; Ruch et al., 2011; Fergus et al., 2015) or “activity steps” (Troped et al., 2008; Nguyen et al., 2013; el Achkar et al., 2016) to report their sampling granularity (Table 1). Activity counts are the sum of the accelerations measured over a selected period (epoch time) (Ruch et al., 2011). Therefore, the studies were grouped into three classes: activity count/step, medium (20–50 Hz), and high (>50 Hz) sampling rate.

**Figure 5:**
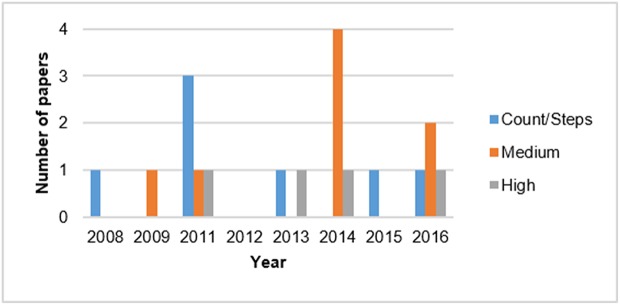
Temporal trend of the sensor sampling rate used for PATD.

Studies show that a higher accelerometer sampling rate provides more relevant information for PATD than data than a lower rate. Furthermore, using a low sampling rate may make it difficult to distinguish transitions between different activities or discriminate characteristics of certain activity types of cyclical, periodic nature (De Vries et al., 2011). The study by De Vries et al. (2011) applied accelerometer data (counts) of epochs of only 1 second, succeeding in classifying the rather distinctive PA types of cycling, walking, and sitting with more that 80% accuracy using two 1D accelerometers. However, the method's performance in discriminating more subtly differing PA types, that is, sitting and standing, going up, and going down the stairs and in differentiating between two different walking speeds and cycling was weak. The authors conclude that in order to detect transitions between activities or characteristics representing activity types of cyclical nature, accelerometer data with a sampling rate >20 Hz are needed. This is confirmed by Figure 5, which suggests that more studies have recently gravitated toward using sampling rates higher than 20 Hz, that is, in the “medium” and “high” categories of Table 1, which is clearly needed to reliably differentiate between multiple types of posture and motion PAs.

##### Sensor placement

**Figure 6:**
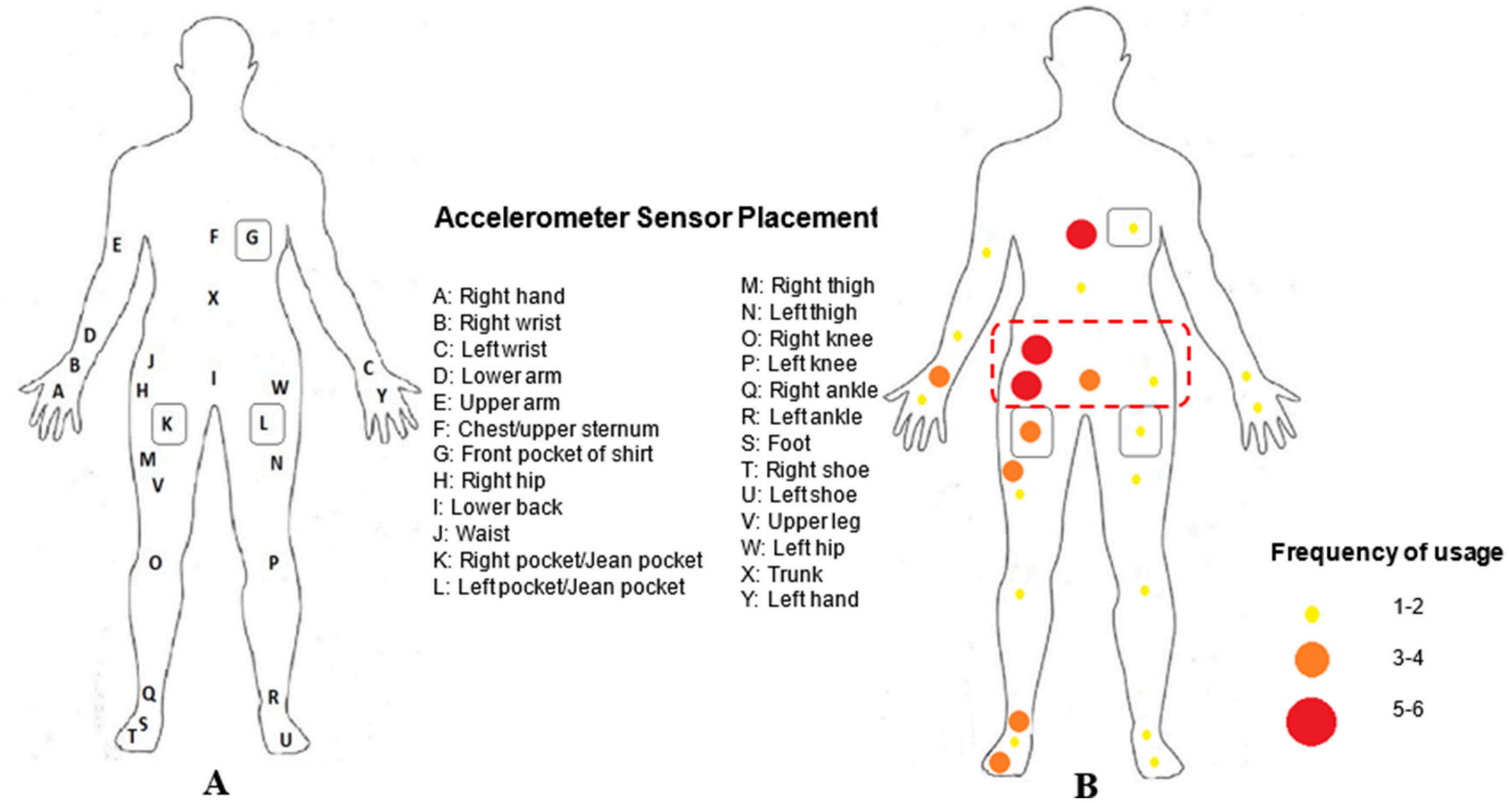
Accelerometer sensor placement: **(A)** Sensor placement; **(B)** Frequency of usage of different sensor placements.

Choosing the sensor placement depends on the type of activities to be identified. Immobility of the device position or wearing comfort for the participants can also play a role in choosing the sensor placement. Figure 6 and Table 2 show the accelerometers' body placement for the included studies. As illustrated, sensors can be placed on the lower part of the body (pants pocket, thigh, leg, ankle, knee, shoe, feet), hands (wrist, upper arm), central part of the body (waist, lower back, hip), or upper part of the body (chest, shirt pocket), or different combinations of the placements mentioned. The central part of the body is used most commonly due to this area being more immobile than the extremities. When only a single accelerometer is used, the most popular sensor location is on the waist (or hip), as this is near the center of the body and can best represent human movement (Liao et al., 2015). Studies have investigated sensor locations for the most accurate classification performance in distinguishing different PA types (De Vries et al., 2011; Skotte et al., 2014; Spinsante et al., 2016), concluding that the positions close to center of the body such as thigh, hip, and pocket can lead to high performance in detecting daily activities such as sitting, standing, walking, running, walking stairs, and cycling.

**Table 2:** Details regarding sensor placement.

**References**	**Sensor placement**	**References**	**Sensor placement**
Fergus et al., 2015	H	Gyllensten and Bonomi, 2011	I, T, U, M, N, F
Spinsante et al., 2016	K	van Hees et al., 2013	B, C, Q, R, H, W, I, E, V
Bayat et al., 2014	A/K/L/Y	el Achkar et al., 2016	T/U, X, M
Godfrey et al., 2011	F	Adaskevicius, 2014	G
Garcia-Ceja and Brena, 2016	F, B, K, J, I, T	Kwak and Lee, 2012	F
Nguyen et al., 2013	J, Q	Troped et al., 2008	H
Skotte et al., 2014	H, M	Ruch et al., 2011	H/W, J
Bonomi et al., 2009	I	Barshan and Yuksek, 2014	B, C, O, P, F
Makikawa and Murakami, 1996	J	Bisio et al., 2012	K/L
De Vries et al., 2011	H, Q/R	Reiss and Stricker, 2011	D, F, S
Shoaib et al., 2014	K, L, E, B, J		

*See the senors placement legend in Figure 6*.

#### Participant Characteristics

This section summarizes the information extracted regarding participant characteristics in terms of age of the study sample and the number of people who participated in the eligible studies.

##### Age

Participants' mobility and PA behavior may differ with age. Children may perform activities more quickly (Ruch et al., 2011), whereas older adults would be expected to be slower. Eligible studies in this review cover different age ranges, from children aged 10–12 (Ruch et al., 2011; Fergus et al., 2015) to older adults with a mean age of 83 (Godfrey et al., 2011). Table 3 illustrates that most studies focused on young people, with only a limited number of studies of older people or children. Using predefined thresholds to distinguish between different physical activities may lead to different findings for the people of different age groups. Therefore the age of the investigated population and of the data used to train the classification model is another key factor for the PATD process.

**Table 3:** Participant characteristics.

**Participant characteristics**		**References**
Age groups	Children (<13 y)	Ruch et al., 2011; Fergus et al., 2015
	Adolescents, young and middle-aged adults (13–55 y)	Troped et al., 2008; Bonomi et al., 2009; Gyllensten and Bonomi, 2011; Reiss and Stricker, 2011; Nguyen et al., 2013; van Hees et al., 2013; Barshan and Yuksek, 2014; Bayat et al., 2014; Shoaib et al., 2014; Skotte et al., 2014
	Older adults (>55 y)	el Achkar et al., 2016
	Adults of all ages	De Vries et al., 2011; Godfrey et al., 2011; Spinsante et al., 2016
Number of participants	<10	Reiss and Stricker, 2011; Bisio et al., 2012; Adaskevicius, 2014; Barshan and Yuksek, 2014; Bayat et al., 2014
	10–30	Troped et al., 2008; Bonomi et al., 2009; Godfrey et al., 2011; van Hees et al., 2013; Shoaib et al., 2014; Skotte et al., 2014; Fergus et al., 2015; el Achkar et al., 2016
	>30	De Vries et al., 2011; Gyllensten and Bonomi, 2011; Ruch et al., 2011; Garcia-Ceja and Brena, 2016; Spinsante et al., 2016

##### Number of participants

Sample size is another key factor for the PATD process. The larger the sample size, the greater the number of activities that can be investigated. Papers were classified into three groups based on the number of participants (Table 3). Surprisingly, most studies were limited to a small sample size (<30 participants). Given a small sample size, only a limited number of activities can be reliably differentiated, and the results may be biased toward the activity behavior of the few selected individuals, with reduced reproducibility. Being able to reliably capture the differences in inter- and intra-individual PA behavior under real-life conditions requires training data sets of large samples.

### Preprocessing Methods

Different preprocessing methods can be applied to prepare the raw accelerometer data for the activity classification process, including filtering, signal segmentation, feature extraction, and feature selection/dimensionality reduction (Table 4). This section summarizes the preprocessing methods used in the included works to answer the second research question.

**Table 4:** Preprocessing methods.

**Preprocessing method**	**Method**		**References**
Filtering	**Butterworth**, median filtering, moving average, FIR		**Godfrey et al., 2011; van Hees et al., 2013; Skotte et al., 2014; el Achkar et al., 2016; Spinsante et al., 2016,** Makikawa and Murakami, 1996; Adaskevicius, 2014; Bayat et al., 2014; Garcia-Ceja and Brena, 2016,
Signal segmentation	Windowing technique	Sliding window	Bonomi et al., 2009; Gyllensten and Bonomi, 2011; Reiss and Stricker, 2011; Bisio et al., 2012; Nguyen et al., 2013; van Hees et al., 2013; Barshan and Yuksek, 2014; Bayat et al., 2014; Shoaib et al., 2014; Skotte et al., 2014; Garcia-Ceja and Brena, 2016
		Activity-based window	el Achkar et al., 2016; Spinsante et al., 2016
Feature extraction	See details in Table 5.		
Feature selection	PCA		Gyllensten and Bonomi, 2011; van Hees et al., 2013; Barshan and Yuksek, 2014
	Clustering		Reiss and Stricker, 2011; Bayat et al., 2014

#### Signal Filtering

Raw acceleration data includes three main components, which are gravity, body acceleration, and noise. The raw signal often contains high-frequency noise that leads to the distortion of the actual signal (Adaskevicius, 2014). In signal processing, a filter removes unwanted components or features from a signal (Smith, 1997). Typical filters used in the eligible studies are for separating signals such as noise removal, or separating the gravitational (DC, low-frequency) component from the body acceleration (AC, high-frequency) component. As Table 4 shows, they include Butterworth (most common), median, moving average and FIR (finite impulse response) filters.

Godfrey et al. (2011) removed signal drift by band-pass filtering the vertical profiles using a 2nd order Butterworth band-pass filter with lower and upper cut-off frequency of 0.15 and 15 Hz, respectively. The elevation was low-pass filtered (Butterworth order 10 filter, 0.1 Hz cutoff) to remove high-frequency noise caused by gait and weather fluctuations that could mask an elevation change in the work of (el Achkar et al., 2016). Skotte et al. (2014) applied a low-pass Butterworth 4th order filter at 5 Hz. Spinsante et al. (2016) used a Butterworth 3rd order filter at 0.3 Hz. van Hees et al. (2013) also applied a Butterworth filter to extract features such as mean and SD of Euclidean norm of the bandpass-filtered acceleration signals (0.2–18, 4th order Butterworth filter) and mean of Euclidean norm of the low-pass-filtered acceleration signals (0.5, 4th order Butterworth filter).

To reduce scattered misclassification, Skotte et al. applied median filtering with a window size of 29 s for cycling and 9 s for the other activities. They found that median filtering improved the overall classification but removed occurrences of short, isolated physical activity types. For example, a walking period shorter than 5 s was not detectable if surrounded by longer periods of standing still (Skotte et al., 2014).

Moving average is a simple filter method for reducing noise. This makes it the premier filter for time domain encoded signals, although it is considered the worst filter for frequency domain encoded signals, with little ability to separate one band of frequencies from another (Smith, 1997). Adaskevicius applied a 5-point moving average filter to reduce the signal noise (Adaskevicius, 2014). The same filter method with 10 points was used for noise reduction by (Garcia-Ceja and Brena, 2016).

Bayat et al. generated a digital low-pass filter with a cut-off frequency of 0.25 Hz. to separate the AC component from the DC component in each time series of accelerometer signals. The AC component is mostly related to the dynamic motion the subject is performing, such as walking or running. On the other hand, the DC component of the acceleration signal is mainly tied to the influence of gravity. The authors concluded that the optimal cutoff frequency in order to exclude the gravity component alone would range from 0.1 to 0.5 Hz (Bayat et al., 2014). Makikawa and Murakami (1996) suggested separating acceleration data into posture data and motion data by the finite impulse response (FIR) filter. They indicated that low-pass FIR filtered data (below 0.1 Hz) contains the subject's posture change, and the rest of the data contains his/her actions. For more information about digital signal processing and filtering, we refer to (Smith, 1997).

#### Signal Segmentation

**Figure 7:**
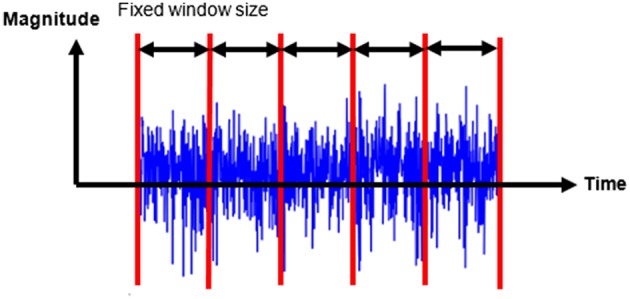
Sliding fixed-size window.

PATD models mostly divide the raw sensor data into smaller segments; classifiers are then applied separately to each window. Windowing techniques are a commonly used signal segmentation approach for PATD, including the sliding window and the activity-based window method. The sliding (or moving) window technique integrates sensor readings over a fixed time (Kozina et al., 2011) as shown in Figure 7. Features are computed per time window and used as input for learning/testing in the classification stage (Kozina et al., 2011). Two approaches are commonly used for data segmentation with sliding windows: the first relies on non-overlapping sliding windows (Bonomi et al., 2009; van Hees et al., 2013; Spinsante et al., 2016), while the seconds uses overlapping sliding windows (Shoaib et al., 2014; Skotte et al., 2014; el Achkar et al., 2016; Spinsante et al., 2016). For example, two consecutive time windows may have 50% of data in common. A range of window sizes have been used in the included studies, ranging from 2 s (van Hees et al., 2013; Shoaib et al., 2014; Skotte et al., 2014) to 1 min (Nguyen et al., 2013). Nonetheless, there was a large inconsistency in deciding which points to choose to segment the data stream. The challenge of the windowing technique is the selection of an adequate segment size for the time window (Shoaib et al., 2014), which decides how often the features are extracted, consequently affecting the PA classifier performance. Moreover, applying a fixed sliding window hinders the exact detection of the activity boundaries, as in natural conditions, the segmentation would rarely correspond with the beginning or end of an activity (Bonomi et al., 2009). Therefore, the overlapping technique may be used to address the problem of boundary ambiguity.

An alternative type of windowing technique is activity-based windowing, where the extracted time windows have to belong to the same activity and are non-fixed (el Achkar et al., 2016; Spinsante et al., 2016). Therefore, activity-based windowing, in contrast to the windowing technique, selects the segment size based on activity transitions, thereby removing boundary ambiguity of acceleration features that might generate a misclassification. However, some studies highlight that fixed-size, overlapping sliding window segmentation is a common approach in medical research, e.g., in patient monitoring, due to the simplicity and ease of interpreting the algorithm (Bersch et al., 2014).

#### Feature Extraction

PATD relies on features that have been extracted from accelerometer signals by transforming the input signals to and from different domains of representation (Figo et al., 2010). Good features should be informative, discriminating between PA types (Barshan and Yuksek, 2014). A *feature space* is formed by the total number of features extracted from the data (Spinsante et al., 2016). Table 5 summarizes the features that were used in the eligible papers, grouped according to the domain in which the features were computed, in this case the *time domain* and the *frequency domain* (Spinsante et al., 2016). Time domain features are typically mathematical or statistical measures derived directly from the sensor data. The window of the sensor data must first be transformed into the frequency domain, normally using a fast Fourier transform (FFT), in order to derive frequency domain features.

**Table 5:** Features used in the PATD process categorized by domain.

**Feature domain**	**Extracted features**
Time domain	Mean, median, average resultant acceleration, min-max, range, variance, SD, coefficient of variation, RMS, interquartile range, nth percentiles, skewness, kurtosis, correlation, angular feature, peak-to-peak distance, cross-correlation, absolute deviation, zero crossings, accelerometer angle, number of peaks, peak amplitude, peak interval, lag-one autocorrelation, autocorrelation sequence
Frequency domain	Dominant frequency, the amplitude of the spectral peak, sum of FFT coefficient, spectral energy, spectral entropy, cross-spectral densities, power of dominant frequency, power spectral density, cross-spectral density, peaks of the DFT

The study of Gyllensten and Bonomi (2011) found that the acceleration features for posture and motion differ greatly in real-life settings from those obtained in laboratory experiments. Also, real-life data showed a higher degree of overlap between features than laboratory data. Hence, determining what the most discriminative features are, acting as input to the PA type classifiers, to achieve the best PATD performance is what makes the feature extraction step challenging (see also the following Feature Selection).A wide range of features have been identified in the literature, depending on the data type from which they are extracted and the target PA types (Spinsante et al., 2016). Thus, it makes little sense trying to compile a simple best list of features. Instead, representative examples are provided below relating features to particular PATD problems. Choosing appropriate features is crucial for detecting desired activity types. For instance, when using a single sensor and the device orientation is not fixed, recognizing certain activity types such as sitting becomes challenging, and the mean values of accelerometer readings do not form discriminative features (Bisio et al., 2012; Barshan and Yuksek, 2014). Extracting orientation independent features such as acceleration magnitude helps (Spinsante et al., 2016). To avoid the orientation problem, a good solution is to estimate the gravity component, which can result in orientation independent features such as the vertical and horizontal components of accelerometer signals as computed in (Adaskevicius, 2014). As noted by Bayat et al. (2014), the average on each axis of a 3D accelerometer over a given time period can serve as a good estimate for gravity component.

To group activities into posture and motion, the SD of the acceleration in the mediolateral axis and SD in the vertical direction were used as discriminating features in (Bonomi et al., 2009) and (Skotte et al., 2014), respectively. (el Achkar et al., 2016) extracted angular velocity from gyroscope readings to distinguish motion from posture by performing step detection. They used the integration metric, which measures the area under the signal curve and is commonly applied to estimate speed from accelerometer signals (Figo et al., 2010). Using the integral of the total magnitude of 3D acceleration, after subtracting the magnitude of static acceleration (gravity), Godfrey et al. (2011) provided an estimate of vertical velocity from a 3D chest-mounted accelerometer to differentiate between walking and the postural transitions of standing to sitting, or sitting to standing. This was achieved by examining the maximum positive and negative peak values of the vertical velocity around the time of a postural transition.

To differentiate between postures, Bonomi et al. (2009) suggested using the cross-correlation between subsequent time intervals of the z-axis of a 3D accelerometer to identify sitting and standing. The scalar product of a chest-mounted 3D accelerometer provided the change of trunk tilt feature, without the need of a gyroscope, helping in separating between postures, i.e., sitting, standing, and lying (Godfrey et al., 2011). Discriminating between sitting and standing postures is challenging using only the inclination of a single 3D accelerometer mounted on the hip, but this feature helps to detect the lying posture (Godfrey et al., 2011; Skotte et al., 2014).

SD in the mediolateral axis and SD of the vertical direction discriminate appropriately in intensity estimation (Godfrey et al., 2011; Skotte et al., 2014), for instance, to differentiate between running and walking (Reiss and Stricker, 2011). Another feature for intensity estimation is the average number of occurrences of peaks, termed average peak frequency (APF), that can better represent high-intensity activities compared to the average time between signal peaks (Bayat et al., 2014). The inclination feature provides a clear-cut separation to discriminate between cycling and horizontal walking and walking stairs. As it is not sufficient to discriminate between level walking and walking stairs, an additional angular feature, the forward/backward angle θ of the thigh was introduced in (Skotte et al., 2014) by using the square root of total magnitude of acceleration and the z-axis component (pointing horizontally forward). Generating a ground slope as an angular feature using frontal and vertical accelerations of a 3D accelerometer during foot-flat position helped to differentiate stairs from uphill/downhill walking in (el Achkar et al., 2016).

#### Feature Selection

As shown in Table 5, a wide variety of features can be extracted from accelerometer data. Feature selection and dimensionality reduction seeks to identify the most informative and best discriminating features in extracted feature vectors, reducing the number of features, thereby decreasing the computational complexity of the classification process and the amount of training data needed for parameter estimations. Two types of techniques, clustering, and principal component analysis, were used for this purpose in the reviewed studies.

##### Clustering

Clustering is a commonly used method to identify the most informative features in feature vectors (Gyllensten and Bonomi, 2011; Spinsante et al., 2016). Relevant features can discriminate clusters, while irrelevant features cannot; therefore clustering can help identify relevant features, thus improving classification efficiency and quality (Bayat et al., 2014).

To illustrate this point, consider the following: Different device orientations may cause acceleration readings, measured for the same activity, to vary between persons. Moreover, different people may perform the same activity in different ways. This variability can lead to substantial differences in the features extracted from accelerometer data. If the feature extraction has been performed well, features belonging to the same activity should form clear clusters in the feature space, while they should be clearly separated if pertaining to different PAs (Spinsante et al., 2016). Likewise, if the clustering algorithm produces homogeneous groups in terms of the activity of their members, this provides a strong indication that a classification algorithm should be successful in differentiating between the different activities (Huynh and Schiele, 2005). Bayat et al. (2014) evaluated different features from the point of view of clustering. The k-means algorithm was used to cluster different features so that the features having the best performance in discriminating PAs could be identified in Reiss and Stricker (2011).

##### Principal component analysis (PCA)

Dimensionality reduction decreases the dimensionality of the feature space to a minimum, thus limiting the computational complexity of the classification process and the amount of training data needed for parameter learning, while still achieving the desired classification performance (Spinsante et al., 2016). PCA also reduces the dimensionality of the feature space to find the most distinguishable features (van Hees et al., 2013). PCA applies an orthogonal transformation converting a set of observations of possibly correlated features into a set of new, linearly uncorrelated features called principal components. Thus, PCA helps to reduce the computational complexity of the PATD process, thereby decreasing the memory and bandwidth requirements for real-time processing on embedded systems (Spinsante et al., 2016). In Barshan and Yuksek (2014), the initially large number of features was reduced from 1170 to 30 through PCA. To visualize the (dis)similarities between data collected in the laboratory and in real-life conditions, two PCA decompositions were performed in Gyllensten and Bonomi (2011): one using different postures and a seconds one using motion activities. Thus, it became apparent that, as mentioned above, the acceleration features for common classes of activities differ greatly in real-life vs. what is measured in the laboratory.

### Physical Activity Type Detection Methods

This section summarizes the eligible studies regarding the number and types of activities detected (Number and type of target PAs), the characteristics of the study setting (Characteristics of data used), as well as the classification methods used and the performance achieved (PA classifiers and their performance). Table S1 provides a concise and complete summary of key parameters [PA types, study setting, classifier(s), performance] for each eligible study.

#### Number and Type of Target PAs

The number of activities represented in the included studies ranges from 2 to 19, with the most common posture and motion activities in real-life conditions including sitting, standing, lying, walking, stairs/non-level walking, running, and cycling, which were introduced as simple activities in the categorization of Spinsante et al. (2016).

#### Characteristics of Data Used

Participants' home, university building, university campus, school playground, or classroom are examples of study settings that have been used for training data collection in the process of PATD. There is large variation in the amount of training data used for PA type classification, with the duration of the data collection ranging from <1 h to 1 week.

#### PA Classifiers and Their Performance

The next step after preprocessing consists of the actual application of a classifier for PATD. Different classifiers have been utilized for PATD in the studies reviewed here, including several types of machine learning (ML) classifiers, fuzzy logic classifiers, rule-based/threshold-based classification, or statistical analysis (Table S1). The degree of complexity of these classifiers varies from simple threshold-based to more advanced algorithms, such as ML algorithms.

Decision trees (DT), a representative of ML classifiers, are the most popular in the included studies when only a single classifier is used. After extracting and selecting appropriate features from the training data the DT algorithm infers thresholds resulting in dichotomous tree splits, based on the derived features for the activity classification. The disadvantage of this method is that if the features are not selected appropriately and include high intra-class variability, the risk of overlaps between values of different activities increases, leading to reduced classification performance due to the relatively simply threshold inference algorithm used (Bonomi et al., 2009). On the other hand, the advantages of this method include low computational requirements and simple implementation (Reiss and Stricker, 2011). As DT can be turned into a graphical representation (i.e., a tree) of the underlying decision rules, they are simpler to understand and interpret than other ML classification methods. Compared to threshold/rule based classifiers (see below), DT have the advantage that the decision structure is inferred automatically by the algorithms and not manually. In the studies that applied DT, an average accuracy above 80% was achieved for detecting more than four different daily living activity types using a small training dataset (Table S1). Activities detected by DT cover all different levels of PA such as sedentary (sitting/standing/lying), moderate (walking), and vigorous (jogging/running) activities (Godfrey et al., 2011; Gyllensten and Bonomi, 2011; Adaskevicius, 2014; Skotte et al., 2014; Garcia-Ceja and Brena, 2016).

Neural network (NN) classifiers, also called artificial neural networks (ANN), take seconds place in the popularity among the reviewed studies that used only a single classifier. However, unlike with DTs, it is more complex and difficult for a user to comprehend how exactly an NN algorithm classifies its inputs. The NN classifiers are powerful approaches that have the potential to be used for detecting different posture and motion activities with high classification performance (Gyllensten and Bonomi, 2011; Ruch et al., 2011; Barshan and Yuksek, 2014; Bayat et al., 2014; Fergus et al., 2015; Spinsante et al., 2016). One of the restrictions of this classifier is the high processing time for the model development, which makes it not a very optimal classifier for a real-time application. Comparing the eligible studies that used this classifier when only a single classifier is used, different architectures of NN behaved differently depending on different numbers and types of activity as well as different study designs (Table S1). When the number of activities increased, the performance of ANN decreased by 14% using two 1D accelerometers (De Vries et al., 2011), whereas in the study of Barshan and Yuksek (2014), NN achieved more than 99% performance in detecting 19 activity types using five 3D IMU and half a day of real-life data. MLP, as a particular type of NN, also gained a very high performance in detecting four activities using unrestricted training data (Fergus et al., 2015).

The k-nearest neighbor (k-NN) classifier is one of the simplest ML algorithms. It assigns a test data point to the class that most of its nearest training data points belong to (Ruch et al., 2011). The restriction of this method is to find the best number of neighbors (k), which is a user-defined constant. Moreover, compared to DTs, k-NN may be slower when a large dataset is processed due to the distance calculation requirements. Using k-NN classification, Adaskevicius (2014) detected daily postures and motion such as sitting and walking at different speeds. He showed how replacing the Euclidean distance by the correlation distance in the acceleration feature could improve the classification performance of k-NN, as the Euclidean distance does not perform well when only a few features play a role in a high-dimensional classification problem.

Fuzzy classifiers are in principle a better method for real-life PATD than conventional approaches, as they can deal with uncertainty in the input data (Kuncheva, 2000). However, defining appropriate fuzzy membership functions and fuzzy rules is challenging (Preece et al., 2009). Kwak and Lee (2012) applied neuro-fuzzy classification, which unlike the common clustering methods, does not consider the borders between neighboring classes to be rigid, but assumes the transitions to be continuous, where an object within the intersection area owns a degree of membership in each class. Seventy Percent classification accuracy was achieved for detecting different walking speeds using accelerometer data only; adding heart rate data the classification accuracy increased to 99.03%. None of the eligible studies used this classifier for detecting postures.

In rule-based classifiers, also called threshold-based classifiers, postures, and motion patterns are detected by manually applying thresholds to the signal of the body worn sensor. The thresholds may be derived from domain or expert-knowledge and used for all subjects. The discrimination between more classes of activities will require a greater number of threshold values. The results of the eligible studies show that this approach performed well in detecting PA using real-life datasets. Postural transition, walking at different intensity levels, lying, sitting, standing, and stairs were successfully distinguished with high classification performance. Of the two studies using threshold-based rules, el Achkar et al. (2016) used multiple sensors, whereas Godfrey et al. (2011) applied a single chest-worn accelerometer. The first approach achieved more than 97% performance in detecting nine different activities using 10 h of data, while the seconds one successfully detected four activities gaining 89% specificity. According to el Achkar et al. (2016) the advantage of their approach is that it is based on rules about biomechanical characteristics of movement, which make it potentially adaptable to populations of different ages as it is not resulting from a training/testing approach and the method can also help in detecting the number of steps. Comparatively, in Godfrey et al. (2011), using the minimal sensor configuration, a reasonable performance in postural transition was obtained.

Using discriminant function analysis, Troped et al. (2008) found that the combination of a GPS and an accelerometer can improve detecting activities such as walking, jogging/running, bicycling, and driving an automobile. Adding GPS can also provide contextual information about the place where the PA is happening, but having only small amounts of data led to a reduced number of detectable activities (Troped et al., 2008).

The variability in the ambulatory assessment specification and preprocessing methods used in the eligible studies makes the comparison between the classifiers difficult. However, our review also covered a few studies that compared different classification methods using same training data set. This helped to achieve some insight regarding the performance of each individual classifier. Spinsante et al. (2016) compared k-NN, NN, and DT, with NN achieving a classification accuracy of 99%, outperforming the other two methods using lab-based data. However, considering additional characteristics such as file size, performance, interpretability and training time all together, DT outperformed the other two. Regarding the time required to train the models, k-NN was the fastest with sub-seconds computation, because it is a “lazy” classifier. The DT classifier was also fast, while the NN method was the slowest (Spinsante et al., 2016).

In the comparative study of (Barshan and Yuksek, 2014), which detected the highest number of posture and motion activities (nineteen) of the studies reviewed, ANN, SVM, and Gaussian mixture models (GMM) achieved the highest performance of above 99% using only half a day of training data. Barshan and Yüksek showed that how using a different validation method or a different software toolbox (PRTools or WEKA) can affect the classification results. For example, using PRTools, it is not possible to initialize important parameters for implementing ANN. Therefore, the performance of ANNs implemented in PRTools does not reflect the true potential of the classifier and is lower compared to when implemented in WEKA. Regarding the validation methods, ANNs and SVMs achieve better results than GMM when applying L1O cross validation, whereas using k-fold cross validation makes the GMM model superior to the other classifiers. Barshan and Yüksek also pointed out that ANNs and SVMs are less sensitive to the overfitting problem compared to the GMMs. However, the GMM method had the advantage of lower computational requirements.

Shoaib et al. (2014) compared 9 classifiers in detecting 8 activity types using different sensors, sensor placements, and extracted features. They showed how these factors of ambulatory assessment specification and preprocessing can play a role in PATD. In their study, the gyroscope performed better than the accelerometer in most cases when either DT or k-NN classifiers were used, especially at the pocket or belt positions using both time and frequency domain features. The results show that the performance of the accelerometer and the gyroscope for recognizing the activities of walking upstairs and walking downstairs, respectively, depend on the body positions, the data features and the classification methods being used.

One of the main challenges of supervised PATD methods is the need for large amounts of labeled data. Garcia-Ceja and Brena (2016), proposed a method which requires only a small amount of labeled data for PATD. The personalized method is based on finding activity similarities between a group of previous users and a target user. Comparing the personalized method with the general model and user-dependent model, the personalized method was best when only a small amount of labeled data was available.

Finally, combining several classifiers is a promising approach, as a meta-classifier can achieve higher classification performance compared to using a single classifier only. Although a meta-classifier will invariably increase the complexity of the classification, it can provide a superior result by combination of simpler individual classifiers (Preece et al., 2009). As shown in Table S1, in all the studies that applied a fusion method, the meta-classifiers outperformed the individual classifiers (Gyllensten and Bonomi, 2011; Ruch et al., 2011; Bayat et al., 2014). However, meta-classifiers also showed weaker performance on real-life datasets compared to using controlled data.

## Discussion

As becomes obvious from the above results, there is a significant variation in the key factors involved in PATD in real-life settings. In this section, we try to distill the insights and lessons that can be gained from our systematic review. We start in General observations with a set of general observations that can be made regarding the overall domain studied. We then continue to discuss the results more specifically regarding two broad and crucial physical activity classes, posture and motion. These two classes of PA not only have different effects on human health, particularly for healthy aging, such as lower levels of functional health and motion activities, a higher risk of falling, and worse cognitive function (Voss et al., 2016), but also directly impact the entire workflow of PATD, including data collection, preprocessing, and PATD methods. Table 6 summarizes the activity types per PA class, as detected by the eligible studies. Detecting Postures presents insights specifically related to distinguishing between different postures, while Detecting Motion Physical Activities reports the key factors regarding differentiating between motion activities. Detecting Posture and Motion Activities provides insights concerning the differentiation of postures from motion activities. Finally, Limitations and Potential Bias briefly points out the limitations of this study.

**Table 6:** Physical activity types.

**Physical activity class**	**Physical activity type**	**Reference**
Posture	Sitting	Troped et al., 2008; Bonomi et al., 2009; De Vries et al., 2011; Godfrey et al., 2011; Bisio et al., 2012; Adaskevicius, 2014; Barshan and Yuksek, 2014; Shoaib et al., 2014; Skotte et al., 2014; Fergus et al., 2015; el Achkar et al., 2016; Garcia-Ceja and Brena, 2016; Spinsante et al., 2016
	Standing	Bonomi et al., 2009; De Vries et al., 2011; Godfrey et al., 2011; Bisio et al., 2012; Barshan and Yuksek, 2014; Skotte et al., 2014; el Achkar et al., 2016; Spinsante et al., 2016
	Lying	Bonomi et al., 2009; Godfrey et al., 2011; Gyllensten and Bonomi, 2011; Reiss and Stricker, 2011; Nguyen et al., 2013; Barshan and Yuksek, 2014; Skotte et al., 2014
	Stationary	Ruch et al., 2011; van Hees et al., 2013
Motion	Walking	Troped et al., 2008; Bonomi et al., 2009; De Vries et al., 2011; Godfrey et al., 2011; Gyllensten and Bonomi, 2011; Reiss and Stricker, 2011; Ruch et al., 2011; Bisio et al., 2012; Kwak and Lee, 2012; Nguyen et al., 2013; van Hees et al., 2013; Adaskevicius, 2014; Barshan and Yuksek, 2014; Bayat et al., 2014; Shoaib et al., 2014; Skotte et al., 2014; Fergus et al., 2015; el Achkar et al., 2016; Garcia-Ceja and Brena, 2016; Spinsante et al., 2016
	Running/jogging	Troped et al., 2008; Bonomi et al., 2009; Gyllensten and Bonomi, 2011; Reiss and Stricker, 2011; Ruch et al., 2011; Bisio et al., 2012; Kwak and Lee, 2012; Nguyen et al., 2013; Adaskevicius, 2014; Barshan and Yuksek, 2014; Bayat et al., 2014; Shoaib et al., 2014; Skotte et al., 2014; Fergus et al., 2015; Garcia-Ceja and Brena, 2016; Spinsante et al., 2016
	Cycling/biking	Troped et al., 2008; Bonomi et al., 2009; De Vries et al., 2011; Gyllensten and Bonomi, 2011; Reiss and Stricker, 2011; Ruch et al., 2011; Nguyen et al., 2013; Barshan and Yuksek, 2014; Shoaib et al., 2014; Skotte et al., 2014
	Non-level walking (upstair/downstair, uphill/downhill)	De Vries et al., 2011; Reiss and Stricker, 2011; Nguyen et al., 2013; Barshan and Yuksek, 2014; Bayat et al., 2014; Shoaib et al., 2014; Skotte et al., 2014; el Achkar et al., 2016; Garcia-Ceja and Brena, 2016; Spinsante et al., 2016
	Other	Troped et al., 2008; Bonomi et al., 2009; De Vries et al., 2011; Gyllensten and Bonomi, 2011; Ruch et al., 2011; Nguyen et al., 2013; Adaskevicius, 2014; Barshan and Yuksek, 2014; Bayat et al., 2014; Fergus et al., 2015; el Achkar et al., 2016; Garcia-Ceja and Brena, 2016

### General Observations

The vastly different use of devices, sensors, sampling rates, and numbers and types of PAs reported in the eligible studies points to a lack of guidelines for PA data collection. Therefore, developing a standardized protocol, or at least a set of best practice guidelines, for data collection in a clearly defined real-life study setting with a concrete and transparent, predefined list of daily living activity types is crucial for future research.

Most studies applied a supervised method for PATD. One of the main challenges of this approach is the need for having labeled data. In relation to the point just made above, we suggest that one or several labeled, documented and openly available reference data sets should be created that cover different age groups and include meaningful types and numbers of transparently defined daily physical activities. Only the availability of common reference data can provide the possibility of comparing existing methods as well as developing improved methods for PATD in real-life settings.

Meanwhile, different validation methods have been applied in the included studies, e.g., training classifiers with one data set and testing with another data set, leave-one-out (L1O) cross validation, and k-fold cross validation. Additionally, the use of different metrics for reporting classification performance, such as overall accuracy, F-score, sensitivity or others is a further issue that makes the comparison between studies difficult.

### Detecting Postures

Among the eligible studies, there are ones that distinguish three postures, namely sitting, standing and lying (Bonomi et al., 2009; Godfrey et al., 2011; Barshan and Yuksek, 2014; Skotte et al., 2014), and ones targeting the two postures sitting and standing (De Vries et al., 2011; Bisio et al., 2012; Shoaib et al., 2014; el Achkar et al., 2016; Spinsante et al., 2016), respectively. There are also studies that combine sitting and standing into one class (sitting/standing) (Gyllensten and Bonomi, 2011; Reiss and Stricker, 2011; Nguyen et al., 2013) or group different types of postures as stationary (Ruch et al., 2011; van Hees et al., 2013).

In general, differentiating between different postures needs to rely on the gravity component of the accelerometer data, particularly gravity direction/inclination.

#### Ambulatory Assessment Specification

As mentioned above, different types of postures such as sitting, standing and lying can be grouped into one class called stationary (Ruch et al., 2011; van Hees et al., 2013; Spinsante et al., 2016). To detect the stationary class, using one single 3D pocket-accelerometer (20 Hz) collected by smartphone (Spinsante et al., 2016), or alternatively applying activity counts/steps from a 1D accelerometer worn on the hip and collected by Actigraph, can be sufficient (Ruch et al., 2011). However, to distinguish between different sub-types of the stationary class such as sitting, standing, and lying, other key factors such as sensor configuration and preprocessing should be considered carefully.

In general, to detect postures, the accelerometer is a very promising sensor as it includes the gravity component. Triaxial accelerometers can better inform a classifier than 1D or 2D accelerometers, and are thus recommended. Sensors such as GPS or foot pressure sensors may be also added to further improve the detection of postures (Nguyen et al., 2013; el Achkar et al., 2016).

A single 3D chest-mounted accelerometer provides a minimal configuration to distinguish not only between sitting, lying and standing but also detect the postural transitions from sitting to standing and from standing to sitting (Godfrey et al., 2011). Similarly, a single placement configuration including a 3D IMU with a foot pressure sensor both embedded in a shoe was found to be sufficient to detect postures and postural transitions (el Achkar et al., 2016). A thigh-mounted 3D accelerometer helps to differentiate between sitting and standing more precisely than an accelerometer placed on the hip (Reiss and Stricker, 2011; Skotte et al., 2014). However, the hip accelerometer data is useful to distinguish the lying posture from sitting/standing (Skotte et al., 2014).

While a single chest-sensor or a single-site shoe embedded sensor configuration may suffice to distinguish the three basic postures of sitting, standing, and lying, more sensors are required to distinguish subtypes of postures. For example, with a five-sensor configuration (knees, wrists, chest), 19 motion patterns and postures including sub-types of lying (lying on the back and on the right side) can be detected (Barshan and Yuksek, 2014).

#### Preprocessing

The raw accelerometer data requires preprocessing, including filtering, signal segmentation, and feature extraction. Low-pass filtering should be used to extract the gravity component of accelerometer signal, which is necessary for detecting postures. Different filters have been applied to reduce noise, including Butterworth and FIR, with Butterworth being the most frequently used.

Regarding features extracted, the gravitational (DC) component of the accelerometer (i.e., signal output <0.5 Hz) allows the assessment of change in position in relation to the gravitational axis (i.e., inclination in degrees). For example, the orientation of the vertical direction of the body with respect to the direction of gravity is the feature to identify lying. The lying posture can be defined as an inclination of the hip accelerometer above 65°(Skotte et al., 2014). Based on the body sensor placement, the calculated inclination can help detecting different postures. For instance, the reason that the thigh accelerometer is able to better detect the sitting posture than the hip/lower-back/waist accelerometer is that the inclination of the hip-mounted accelerometer does not differ significantly between the standing position and upright sitting. The inclination of a thigh accelerometer gives the angle between the vertical line and the thigh axis, which can more precisely differentiate between sitting and standing. The trunk tilt feature extracted from a chest sensor can distinguish between lying and sitting/standing (Godfrey et al., 2011). Lying can also be detected with high performance using the peak absolute value of a chest-mounted accelerometer, as it has a different upper body orientation compared to other activities (Reiss and Stricker, 2011). The cross-correlation between subsequent time intervals of the antero-posterior lower-back acceleration can identify sitting and standing (Bonomi et al., 2009). Similarly, the intensity of a vertical accelerometer mounted on the waist helps distinguishing between sitting and standing (Nguyen et al., 2013). The total force (TF) parameter can be calculated using foot pressure sensors embedded in a shoe by considering a person's body weight, which is helpful to distinguish sitting and standing and also their transitions (el Achkar et al., 2016).

#### Physical Activity Type Classification Methods

Misclassification issues may be observed in differentiating between sitting, standing and lying using real-life datasets (Bonomi et al., 2009; De Vries et al., 2011; Gyllensten and Bonomi, 2011; Reiss and Stricker, 2011; Skotte et al., 2014) as, for example, a leaning posture might be more frequent in real-life compared to the controlled condition. Also, in real-life settings, there is significant inter-individual variability in postural states (Gyllensten and Bonomi, 2011). Therefore, a larger number of subjects from different age cohorts are required to investigate these effects. Using the threshold-based approaches, it is important to consider the precise location and fixation of the sensors, as the sensor orientation can affect the recorded data and alter the expected results (Adaskevicius, 2014). A chest-mounted accelerometer or an IMU together with a foot pressure sensor embedded in the shoe can result in high threshold-based classification accuracy with a minimal sensor configuration for detecting postures (Godfrey et al., 2011; el Achkar et al., 2016).

### Detecting Motion Physical Activities

Among the motion activities, active modes of transport such as walking, cycling/biking, and jogging/running contribute to reduced risk of physical and mental health problems (Physical Activity Guidelines Advisory Committee, 2008). Some of the included studies detected all these active modes of transport (Troped et al., 2008; Bonomi et al., 2009; Gyllensten and Bonomi, 2011; Reiss and Stricker, 2011; Ruch et al., 2011; Nguyen et al., 2013; Barshan and Yuksek, 2014; Shoaib et al., 2014; Skotte et al., 2014). Almost all the eligible studies detected the walking activity, while some also considered different speeds of walking (Reiss and Stricker, 2011; Kwak and Lee, 2012; Adaskevicius, 2014; Bayat et al., 2014). Running/jogging is the second commonly detected motion activity, followed by cycling/biking. Almost half of the included studies detected non-level walking activities such as walking downstairs/upstairs or walking downhill/uphill (De Vries et al., 2011; Reiss and Stricker, 2011; Nguyen et al., 2013; Barshan and Yuksek, 2014; Bayat et al., 2014; Shoaib et al., 2014; Skotte et al., 2014; el Achkar et al., 2016; Garcia-Ceja and Brena, 2016; Spinsante et al., 2016).

In general, motion activities mostly involve the movement of the whole body. And therefore, differentiating between different motion activities needs to make use of the body motion component of the accelerometer data.

#### Ambulatory Assessment Specification

Similar to the process of posture detection, for recognizing the active modes of transport, a multi-axis accelerometer is a promising sensor to use. The results indicate that for detecting walking, cycling, and running, the accelerometer performs better than other sensors such as a gyroscope regardless of sensor placement, the feature set, and the classifier used (Shoaib et al., 2014). However, the gyroscope is also able to detect these activities reasonably well. Linear acceleration (the AC component) can also be used for detecting these motion activities. A magnetometer also achieves reasonable performance when direction-insensitive features such as variance, zero crossings and root mean square values are used (Shoaib et al., 2014). Additional sensors such as GPS (Troped et al., 2008; Nguyen et al., 2013) and heart rate sensors (Reiss and Stricker, 2011; Kwak and Lee, 2012) can improve motion detection. (Troped et al., 2008; Nguyen et al., 2013) are two of very few studies using an accelerometer and GPS data in combination, despite the high potential that GPS could have in providing spatial contextual information that could further inform the detection of motion PAs. However, aiming for a minimal sensor configuration, which is preferable for real-life PA monitoring, a single waist-mounted 3D accelerometer is sufficient for detecting walking, running, and cycling (Bonomi et al., 2009; Gyllensten and Bonomi, 2011). Conversely, to reliably distinguish non-level walking, additional sensors such as a barometer (el Achkar et al., 2016) or a 3D thigh-mounted accelerometer may be required (Skotte et al., 2014). The target motion activities determine the sensor placement. For instance, a sensor placed on the wrist is preferable when trying to distinguish daily-life activities with similar lower-body, but significantly different upper-body movement (Reiss and Stricker, 2011). For instance, using a hand (wrist, upper arm, hand) accelerometer, by extracting the periodic pattern of arm swinging, motions such as walking at different speeds and running can be detected (Reiss and Stricker, 2011).

#### Preprocessing

High-pass filtering helps to isolate the body motion of the acceleration signal (Bayat et al., 2014). Depending on the type and placement of the sensor, different informative features for detecting motion activities can be extracted. Representative distinctive features for differentiating motion activities are provided below.

The forward/backward acceleration in hand/pocket placement can represent the periodic behaviors of walking at different speeds, running, and non-level walking, but with distinctive patterns (Bayat et al., 2014). Applying the same sensor placement, the average number of occurrences of peaks in each signal window of the accelerometer instead of average time between peaks is also a useful feature for recognizing high-intensity activities (Bayat et al., 2014). Using a 3D accelerometer, the variance or SD in different axes is an indicator of different motion activities. For example, using a lower-back mounted accelerometer, a high value of the SD of the acceleration in the vertical direction of the body is indicative of running, while the SD in the antero-posterior and the vertical direction can be used to discriminate walking and cycling (Bonomi et al., 2009). The SD of the vertical axis of a 3D thigh-mounted accelerometer can differentiate between running and walking, as well as between postures and other motion activities. Using the same sensor placement, the inclination feature can discriminate between cycling and walking stairs. The forward/backward angle of the thigh is distinctive for walking/running and non-level walking (Skotte et al., 2014). Using the frontal and vertical accelerations of a shoe-embedded 3D accelerometer can provide an informative feature differentiating stairs from walking uphill/downhill (el Achkar et al., 2016).

#### Physical Activity Type Detection Methods

The type of sensor as well as sensor parameters used can affect the classification performance. For example, a simple ANN model based on two 1D accelerometers may not be successful in discriminating between two self-paced speeds of walking and cycling using accelerometer counts (De Vries et al., 2011). Conversely, using a sensor configuration comprising 5 IMU, an SVM classifier was able to differentiate between 19 motion activities and postures with high accuracy, followed by an ANN using the WEKA software (Barshan and Yuksek, 2014). However, using a different software toolbox (PRTools), the GMM model took the leading role applying repeated random sub-sampling (RRSS) and k-fold cross-validation as validation methods; applying L1O cross validation, ANNs and SVMs stayed superior using the PRTools toolbox (Barshan and Yuksek, 2014). These results show that even the software and validation methods used may have an influence on the classification performance that can be achieved. Finally, the results of Bonomi et al. (2009) indicate that applying the minimum sensor configuration of a single lower-back 3D acceleration, a DT classifier is able to successfully distinguish between different motion activities with an accuracy of 93%.

### Detecting Posture and Motion Activities

#### Ambulatory Assessment Specification

Most human PA routines may be described by the motion activities of walking, cycling, running, and the postures of sitting, standing, and lying. It is thus important to be able to distinguish between these activities in the real-life PATD process (Reiss and Stricker, 2011). Moreover, from the clinical point of view, it is particularly crucial for PA behavior monitoring in the healthy aging context to discriminate postures such as sitting and standing from motion types such as walking (el Achkar et al., 2016).

While a dual accelerometer position is recommended for reliable PA distinction, particularly for postures (De Vries et al., 2011; Gyllensten and Bonomi, 2011; Reiss and Stricker, 2011), there is evidence asserting the validity of using a single 3D accelerometer with (el Achkar et al., 2016) or without (Godfrey et al., 2011) additional sensors on a single position for accurate activity classification of postures and transitions between them. A 3D accelerometer provides more information compared to 1D and 2D accelerometers. Using a 1D or 2D accelerometer will increase the number of required sensors (Troped et al., 2008; De Vries et al., 2011; Ruch et al., 2011; Nguyen et al., 2013; Fergus et al., 2015) for reliable PATD. To detect all the basic postures and motion activities in daily life, using a single-position system was shown to be problematic (Gyllensten and Bonomi, 2011). Two 3D accelerometers mounted on the thigh and hip, respectively, was shown to distinguish with a high classification accuracy of more than 95% the main types of postures and motions, that is, sitting, standing, lying, walking, cycling, running, and non-level walking (Skotte et al., 2014).

Additional sensors such as barometer or force sensing are helpful for detecting non-level walking and postures (Skotte et al., 2014; el Achkar et al., 2016), while GPS and heart-rate sensors improve the detection of motion activities (Troped et al., 2008; Kwak and Lee, 2012; Nguyen et al., 2013). Sensors without gravity component, such as a gyroscope, and linear acceleration perform poorly in differentiating postures such as sitting and standing. However, adding an accelerometer with a vertical axis can address this classification problem (el Achkar et al., 2016). Conversely, the classification performance of these sensors is comparable or sometimes better than a 3D accelerometer in recognizing motion activities (Shoaib et al., 2014).

Devices such as the Actigraph provide activity counts. Activity counts are the sum of the accelerations measured over a selected period (epoch time) of e.g., 1 s (Ruch et al., 2011). Usually the activity counts are filtered and preprocessed accelerometer data. The activity counts from 1D accelerometers may not accurately detect activity transitions or reveal the cyclical pattern of motion activities such as cycling. Therefore, using raw accelerometer data with more than 20 Hz sampling rate is recommended (De Vries et al., 2011). There are also studies that applied higher sampling rates of more than 20 Hz (Bonomi et al., 2009; Gyllensten and Bonomi, 2011; Adaskevicius, 2014; Barshan and Yuksek, 2014; Shoaib et al., 2014; Skotte et al., 2014; Garcia-Ceja and Brena, 2016; Spinsante et al., 2016) or more than 50 Hz (Reiss and Stricker, 2011; van Hees et al., 2013; Bayat et al., 2014; Garcia-Ceja and Brena, 2016), while (Shoaib et al., 2014) indicated that 50 Hz can be a sufficient sampling rate to recognize daily PAs.

#### Preprocessing

The high-frequency component of the acceleration signal, the AC component, is mostly related to the dynamic motion activities such as walking or running, while the low-frequency component of the acceleration signal, the DC component, is mainly tied to the influence of gravity, which plays an important role for postures. To extract the gravity component a low-pass filter with a cutoff frequency in the range from 0.1 to 0.5 Hz can be applied. To obtain the AC component, the low-pass filtered data can be subtracted from the original data (Bayat et al., 2014).

When using windowing techniques for signal segmentation the window size should be carefully selected. In real-life settings, activities are not happening continuously and it is common to have many short bouts of activities. Therefore, a signal segment may comprise several activities. Choosing a small window size may be useful in detecting activity transitions but may lead to a reduction of the classification accuracy. For example, using a 20 Hz 3D accelerometer, window sizes of 0.4, 0.8, 1.6, 3.2 s achieved lower classification accuracy in discriminating motion activities and postures compared to window sizes of 6.4 or 12.8 s (Bonomi et al., 2009). Conversely, large windows react more slowly to activity changes but provide better protection against misclassification (Bonomi et al., 2009; Bisio et al., 2012). The window size should be large enough to include the signal signature of motion activities such as walking, cycling, and running to capture several cycles of the corresponding acceleration data (van Hees et al., 2013; Bayat et al., 2014; Skotte et al., 2014). Overlapping windowing can also be useful to reduce information loss at the edges of the signal window (Bonomi et al., 2009).

Regarding feature extraction, it is important to note that generally, the acceleration features for postures and motion activities differ greatly in real-life settings from those obtained in laboratory experiments. In particular, there is a higher degree of overlap between the empirical distributions of features generated in real-life settings than from laboratory data (Gyllensten and Bonomi, 2011).

Above, we already discussed features that are useful to discriminate different posture types (Preprocessing) and different types of motion activities (Preprocessing), respectively. If the task is to differentiate between postures and motions, it is common to use features that represent the variation in the acceleration signal. For example, high values of the SD of the waist-acceleration in the mediolateral direction are an indicator of motion activities, whereas low values of this feature are indicative of postures (Bonomi et al., 2009). When a subject is performing motion activities, the acceleration signal is oscillating in a cyclical pattern, with varying peak amplitude, but constant peak interval. The higher the peak amplitude is, the more intense the motion activity (Makikawa and Murakami, 1996). Conversely, when the subject remains in a postural state, the accelerometer signal is not oscillating. The acceleration peaks can be recognized by step detection and help to distinguish between postures and several motion activities. The angular velocity from a gyroscope can be used to detect steps based on the toe off (TO) instant (el Achkar et al., 2016). The number of steps or the GPS speed (Troped et al., 2008; Nguyen et al., 2013) are informative features for discriminating between postures and motion. The vertical velocity estimated from a 3D chest-mounted accelerometer also helps to differentiate these two classes of PA (Godfrey et al., 2011).

#### Physical Activity Type Detection Methods

Different classifiers were used to detect posture and motion activities including several ML classifiers (Bonomi et al., 2009; De Vries et al., 2011; Gyllensten and Bonomi, 2011; Reiss and Stricker, 2011; Ruch et al., 2011; Bisio et al., 2012; Nguyen et al., 2013; van Hees et al., 2013; Adaskevicius, 2014; Barshan and Yuksek, 2014; Bayat et al., 2014; Shoaib et al., 2014; Skotte et al., 2014; Fergus et al., 2015; Spinsante et al., 2016), fuzzy logic classifiers (Kwak and Lee, 2012), rule-based/threshold-based classification (Godfrey et al., 2011; Shoaib et al., 2014; el Achkar et al., 2016), or statistical analysis (Troped et al., 2008). Almost all of the studies detected both motion activities and postures, with the exception of two studies (Kwak and Lee, 2012; Bayat et al., 2014), which only detected motion activities. There is a high variation in the number, type and body placement of the sensors, preprocessing methods used, characteristics of the training dataset, validation methods, number and type of target PAs, and the classification toolbox applied in the eligible studies, all of which could alter the classification result of a particular PATD exercise. Therefore, it is difficult to make a concrete conclusion as to what is the best classifier for detecting postures, motion activities, or both PA classes. For instance, in one study DT (a rather simple classification method) was able to detect all the motion activities and postures mentioned above with a high accuracy using two 3D accelerometers (30 Hz) on the hip and thigh (Skotte et al., 2014). However, using a single 3D waist/lower back accelerometer (20 Hz) in a different study decreased the DT classification performance for postures such as sitting and standing (Bonomi et al., 2009). This suggests that the performance of the classifier used responds to the design of the ambulatory assessment specification and the preprocessing operations used.

In general, as explained in detail in PA classifiers and their performance, different classifiers can have different strengths and weaknesses, depending on the classification problem at hand. Considering not only the classification accuracy alone, but also additional criteria such as the amount of training data required, computational performance, interpretability, and required training time (Spinsante et al., 2016), all of which are important factors for real-life PATD, the DT classifier seemed to be the most promising approach for reliably detecting basic classes of postures and motion activities.

### Limitations and Potential Bias

To conduct the systematic literature review, only four databases were searched, which may have kept other relevant studies contained in other databases from being included. Based on the inclusion criteria, only literature in English was included; studies in other languages were not considered. The particular focus on approaches that performed the data collection in real-life conditions may have led to excluding potentially advanced laboratory-trained algorithms that have not been validated with real-life datasets so far. However, this study had two strengths: journal articles and conference papers that were derived from four comprehensive databases were rigorously screened based on the eligibility criteria, and the articles included were carefully analyzed in a standardized way.

## Conclusion

This systematic review performed an analysis of the literature since 1990 to present key factors regarding current methods of physical activity type detection (PATD) using accelerometer data collected in real-life settings. In general, the eligible studies showed that assessing daily real-life PA has seen major advances in the past decade due to progress in portable sensor technology, in particular relating to accelerometry. The results of this review were structured according to the three main stages of the PATD process, data collection, preprocessing, and PATD methods, and led to the following major findings:

### Data Collection

Actigraph, which supports continuous tracking over several days, was the most commonly used commercial device. Nonetheless, smartphones have gained popularity in recent years, mainly due to their ubiquitous use in daily life and their multiple sensors. However, compared to dedicated devices, smartphones have a shorter battery life and may suffer from uneven sampling rates. In terms of sensor type, 3D accelerometers are now most often used, typically with sampling rates (considerably) higher than 20 Hz in real-life settings. For sensor placement, locations close to the central part of the body, such as waist and hip, are most common. Most studies used small sample sizes between 10 and 30 participants, but given the increasing availability and affordability of mobile sensing devices, larger sample sizes are becoming increasingly feasible.

### Preprocessing

Preprocessing of the accelerometry data is crucially important for the accuracy that can be achieved in the subsequent PA type classification stage. The eligible studies did not show great variation regarding signal filtering and segmentation: Butterworth was the most commonly used filter, followed by moving average and median filters. Low-pass filtering can extract the gravity (DC) component, which is important for differentiating postures, while high pass filtering is useful to derive the body acceleration (AC component). In signal segmentation, windowing techniques were often employed, either using a fixed-size sliding window or an activity-based window. In feature extraction, a great diversity of features have been used across the various studies, both in the temporal and in the frequency domain, with the choice of features dependent on the PA types investigated. Some publications made recommendations as to the discriminating qualities of certain features. Nonethless, feature selection and dimensionality reduction, as a final preprocessing step, are crucial to obtain an informative feature set; clustering techniques as well as PCA were commonly employed for this purpose.

### PATD Methods

The most typical PA types investigated were sitting, standing, and lying from the posture PA class and walking, non-level walking, running, and cycling from the motion PA class. There was a large variation in the amount of training data used for the PA type classification. A wide range of classifiers have been employed, both as individual classifiers and as meta-classifiers, combining different individual methods to achieve the best possible classification result. Among the individual classifiers, decision trees were most commonly used, followed by neural networks. Combining classifiers to meta-classifiers was shown to be a promising approach. Indeed, the meta-classifiers outperformed the individual classifiers in all studies that applied a combined method.

### Posture and Motion Physical Activities

Posture and motion activities are two important PA classes that not only have different effects on human health, but also require different PATD designs and workflows. The type of target PA and whether it is from a posture or motion class determines the design of the data collection, the preprocessing operations required, and eventually the classification performance that can be achieved. The gravity component of the accelerometer data, particularly the gravity direction/inclination, needs to be established in order to discriminate between different postures. A single 3D chest-mounted accelerometer provides a minimal configuration to discriminate between the most common postures (sitting, lying, and standing) and their transitions, but more sensors are required to distinguish further subtypes of postures. In contrast to postures, motion activities involve the movement of the whole body and thus differentiating between them requires the use of the body acceleration (AC) component of the accelerometer data. A single waist-mounted 3D accelerometer is sufficient for detecting the most common motion activities (walking, running, and cycling). Again, in order to reliably distinguish more fine-grained motion activities, additional sensors in different placements are required.

Despite the significant progress made over the past years, it remains difficult, if not impossible, to compare the performance of the various proposed methods. A transparent performance comparison would require, most importantly, an agreed set of PA types that could be used for benchmarking; labeled, fully documented, and openly available reference datasets representing the selected PA types; and an agreement on a set of performance metrics, evaluation and reporting protocols. This would enable development of broadly applicable guidelines and recommendations, facilitating robust progress both concerning PA type detection methods and their application in a growing number of application domains, including sports medicine, healthy aging, smart homes, and ubiquitous computing. Finally, while most work reviewed in this paper relied on dedicated accelerometer devices, there is clearly a trend toward the use of smartphones, as these can support a wide range of apps on top of accelerometry. Future research should therefore look into the work of relevant domains, in particular pervasive computing.

## Author Contributions

HA developed the idea of the manuscript. All authors contributed to the design. HA assessed all titles and abstracts and all full-text articles. Decisions to accept or reject a paper were agreed among all authors. HA wrote the first draft. All authors contributed to internal manuscript revision, read, and approved the submitted version.

### Conflict of Interest Statement

The authors declare that the research was conducted in the absence of any commercial or financial relationships that could be construed as a potential conflict of interest.

## Abbreviations

PATD: Physical activity type detection.
