# Supplementary material for: The Key Factors in Physical Activity Type Detection Using Real-Life Data: A Systematic Review
Source: Front Physiol. 2019 Feb 12;10:75. doi: 10.3389/fphys.2019.00075 (PMC6379834; doi:10.3389/fphys.2019.00075)
Supplement: Supplementary file 1 [file Table_1.docx]

Supplementary Material

**The Key Factors in Physical Activity Type Detection Using Real-Life Data: A Systematic Review**

**Hoda Allahbakhshi^1*^ , Timo Hinrichs^2^, Haosheng Huang^1^, Robert Weibel^1^**

*** Correspondence: Hoda Allahbakhshi**: hoda.allahbakhshi@geo.uzh.ch

**Table S1** Physical activity type detection methods and their characteristics. Note that for the description of the classification performance, the terms used are found in the corresponding publication.

| **Method** | **Types and number of detected activities** | **Study design and total data volume** | **Number of participant (No.)**  **Age of participants**  **Duration of protocol (T)** | **Classifier & reference** | **Classification performance** | |
| --- | --- | --- | --- | --- | --- | --- |
|  |  |  |  |  | **Lab/controlled + outdoor protocol** | **Real-life protocol** |
| Individual machine learning classifiers | sitting, standing, lying, walking, running, cycling, stairs (7) | - Standardized protocol (8.5 hours)  Outdoors: Self-speed walking, running, cycling  - Real-life: Sitting (5.8 days) | No. = 17, Age ~ 34 ± 11 years | (Skotte et al. 2014)  DT | Sensitivity: >95%  Specificity: >99% | For sitting:  sensitivity: 98.2%  specificity: 93.3% |
|  |  |  | Training dataset:  T = 30 min |  |  |  |
|  |  |  | Test dataset for sitting:  T = 9 hours |  |  |  |
|  | walking, burst displacement, vehicle, running, sitting/standing, cycling etc. (8) | - 2 outdoor controlled protocols (> 1 day)  - Real-life (1.4 days) | Total No. =41, Age ~ 25 years | (Nguyen et al. 2013)  DT | Not reported | Not reported |
|  |  |  | Training dataset:  No.=12, T = Unknown |  |  |  |
|  |  |  | Validation dataset I (controlled):  No. = 12, T ~ 45 min |  |  |  |
|  |  |  | Validation dataset II (real-life):  No. = 17, T = 2 hours |  |  |  |
|  | walking normal, running, cycling, Nordic walking, lying, sitting/standing, other (ascending, descending stairs etc.) (8) | Controlled protocol (8 hours)  Outdoors: walking at diff. speed, running, cycling, partially other | - No. = 8  - Age = 27.88 ±2.17 years  - T ~ 1 hour | (Reiss & Stricker 2011)  DT | Overall performance: 86.80% |  |
|  | sitting, standing, walking, running (4) | Real-life (17 hours) | - No. = 4  - Age = Unknown  - T ~ 4 hours | (Bisio et al. 2012)  DT |  | Overall accuracy  Framed based: 98%  Window based: 88.24% |
|  | lying, sitting, standing, dynamic standing, walking, running, cycling (7) | Controlled protocol (20 hours)  Outdoors: walking, running, cycling | - No. = 20  - Age = 29 ± 6 years  - T ~ 1 hour | (Bonomi et al. 2009)  DT | Overall accuracy: 93% |  |
|  | sitting, standing, ascending stairs, descending stairs, walking, cycling etc. (9) | Controlled protocol (1.5 days)  Outdoor: walking, cycling | - No. = 49  - Age = 38 ± 11 years  - T = 45 min | (De Vries et al. 2011)  ANN | Correctly classified 5 activities: 83.0%  Correctly classified 9 activities: 69.1% |  |
|  | drawing, free play, jogging, and walking (4) | Real-life (16 hours) | - No. = 16  - Age = 11.4 ± 0.3 years  - T ~ 1 hour | (Fergus et al. 2015)  MLP |  | Overall accuracy:  96% |
|  | low speed walking, high speed walking, sitting, shoulder lifting, squatting, jumping (6) | Real-life (50 minutes) | - No. = 5  - Age = Unknown  - T ~ 10 min | (Adaskevicius 2014)  k-NN |  | Overall accuracy: 78.9% |
|  | walking, non-walking (2) | - Structured activity protocol (16 mins) Outdoor: cycling  - Producing 4 simulated real-life datasets using hypothetical real-life scenarios and 1 lab dataset using the protocol dataset | - No. = 28  - Age ~ 21–53 years  - T = 2 min of data for each of 8 classification models | (van Hees et al. 2013)    Binary classification | Sensitivity: 93% Specificity: 98% | Accuracy:  82–89% |
|  | | | | | | |
| Fuzzy Logic | normal walking, brisk walking,  slowest running, jogging (4) | Real-life (Unknown) | - No.= Unknown  - Age = Unknown  - T = 206 samples | (Kwak & Lee 2012)  TSK-ANFC |  | Classification performance:  Accelerometer only: 70.37%  Accelerometer & HR: 99.03% |
|  | | | | | | |
| Rule-based / threshold-based classifiers | sitting, standing, level walking, upstairs, downstairs, uphill, downhill, elevator up, elevator down (9) | Real-life (10 hours) | - No. = 10  - Age = 65–75 years  - T ~ 1 hour | (el Achkar et al. 2016)  Expert/ threshold-based |  | Overall accuracy: 97.41% |
|  | walking, lying, standing to sitting transition, sitting to standing transition (4) | Controlled protocol: young people (279 step counts)  Real-life: elderly (253 steps) | Training dataset:  - No. = 10  - Age= 23.7 ± 2.2 years  - T = 16–35 steps | (Godfrey et al. 2011)  VESPA | sensitivity: 86% specificity: 92% | sensitivity: 83%  specificity: 89% |
|  |  |  | Test dataset:  - No. = 10  - Age = 77.2 ± 4.3 years  - T = 11–29 steps |  |  |  |
|  | | | | | | |
| Statistical analysis | walking, jogging/running, bicycling, inline skating,  driving automobile (5) | Real-life  Calibration protocol: ~4.6 hours  Validation I: ~7.1 hours  Validation II: ~3.3 hours | No. = 10, Age ~23–51 years  T = 10 min for each activity | (Troped et al. 2008)  SAS PROC DISCRIM |  | Correct differentiation rates:  validation 1: 98%  validation 2: 91% |
|  |  |  | Training data:  Total T = 286 min |  |  |  |
|  |  |  | Validation data I:  Total T = 430 min |  |  |  |
|  |  |  | Validation data II:  Total T = 200 min |  |  |  |
|  | | | | | | |
| ­­­­­­­Combined classifiers | stationary, walking, running, jumping, floor exercise, biking, horseback riding, crawling (8) | Real-life (7 days) | No. = 41, Age ~10–12 years  Total T = 7 days | (Ruch et al. 2011)  MV, k-NN, NDDf, DT |  | Recognition rate of MV & k‑NN: 67% |
|  |  |  | Training data:  No. = 21, T ~ 570–573 counts/min |  |  |  |
|  |  |  | Test dataset:  No. = 20, T ~ 526-675 counts/min |  |  |  |
|  | lying, sitting/standing, dynamic/transitions, walking, cycling (6) | Controlled protocol (5 hours)  Training data (14.5 hours)  Outdoors: walking, cycling, running  Real-life (10.5 hours) | Training data:  No. = 52, Age ~29.2 ± 6.7 years  Total T = 14 h and 34 min | (Gyllensten & Bonomi 2011)  MV  DT, NN, SVM | Overall accuracy of MV:  controlled: 95.1%  training: 95.9% | Overall accuracy of MV:  75.7% |
|  |  |  | Laboratory validation data:  No. = 20, Age ~30±9 years  Total T = 4 h and 59 min |  |  |  |
|  |  |  | Real-life validation data:  No. = 20, Age ~30±9 years  Total T = 246 hours and 28 min |  |  |  |
|  | running, slow-walk, fast-walk, aerobic dancing, stairs-up, stairs-down (6) | Real-life (13 mins) | - No. = 4  - Age = 29–33 years  - T ~ 3–4.5 min | (Bayat et al. 2014)  Average of probabilities, MLP, SVM, RF, LMT, Simple Logistic, Logit Boost |  | Overall accuracy of average of probabilities (MLP, Logit Boost, SVM): 91.15% |
|  | | | | | | |
| Comparing different classifiers | walking, running, sitting, standing, jogging, biking, walking upstairs, walking downstairs (8) | Real-life, two settings: university building (4.6 hours), outdoors: biking | - No. = 10  - Age = 25-30 years  - T ~ 21-28 min | (Shoaib et al. 2014)  BN, NB, LSM, LR, k-NN, PART, NNGE, DT (J84, RF) |  | Not reported |
|  | sitting, standing, lying, ascending stairs, descending stairs, standing, walking, running, cycling, rowing, jumping, playing basketball etc. (19) | Real-life (12.5 hours) | - No. = 8  - Age = 20-30 years  - T ~ 95 min | (Barshan & Yuksek 2014)  NB, ANNs, DBC, DTs ((J48-T), NB trees (NB-T), (RF-T)), GMMs, SVMs. |  | Correct differentiation rates  ANNs: 99.2%  SVMs: 99.2%  GMM: 99.1% |
|  | walking, jogging, ascending stairs, descending stairs, sitting, standing (6) | Controlled protocol (15 hours)  Real life (1.75 days)  DT when trained with generalized real-life walking data for detecting 6 activities and  improved DT trained with generalized real-life sitting data for detecting 2 activities | Training lab dataset:  - No. = 36  - Age ~23–51 years  - T = 25 min | (Spinsante et al. 2016)  DT, k-NN, NN, | Controlled data  NN: weighted average F-score (WA-F-score) for detecting 6 activities = 0.968 | DT trained with generalized real-life data  (WA-F-score)  for detecting 6 activities: 0.923,  (WA-F-score)  for detecting 2 activities: 0.988 |
|  |  |  | Training real-life dataset:  - No. = 563  - Age ~23–51 years  - Total T = 42 h |  |  |  |
|  | walking, jogging, upstairs, downstairs, standing, sitting etc. (15) | Real-life and Lab (~26000 instances) | Dataset 1:  No.=15, Age ~27-35 years  T = 8506 instances | (Garcia-Ceja & Brena 2016)  General model, user-dependent model, personalized model (RF) |  | Accuracy of personalized model:  D1: > 80%  D2: > 70%  D3: > 80%  D4: > 90%  D5: > 80% |
|  |  |  | Dataset 2:  No.=16, Age ~19-81 years  T = 2807 instances |  |  |  |
|  |  |  | Dataset 3:  No.=36, Age ~23–51 years  T = 5418 instances |  |  |  |
|  |  |  | Dataset 4:  No.=21, Age ~19-48years  T = 7352 instances |  |  |  |
|  |  |  | Dataset 5:  No.=4, Age ~ Not reported  T =1855 instances |  |  |  |
